# Multiplexed Digital mRNA Profiling of the Inflammatory Response in the West Nile Swiss Webster Mouse Model

**DOI:** 10.1371/journal.pntd.0003216

**Published:** 2014-10-23

**Authors:** José Peña, Jessica A. Plante, Alda Celena Carillo, Kimberly K. Roberts, Jennifer K. Smith, Terry L. Juelich, David W. C. Beasley, Alexander N. Freiberg, Montiago X. Labute, Pejman Naraghi-Arani

**Affiliations:** 1 Lawrence Livermore National Laboratory, Livermore, California, United States of America; 2 Department of Pathology, University of Texas Medical Branch, Galveston, Texas, United States of America; 3 Sealy Center for Vaccine Development, University of Texas Medical Branch, Galveston, Texas, United States of America; 4 Department of Microbiology and Immunology, University of Texas Medical Branch, Galveston, Texas, United States of America; 5 Center for Biodefense and Emerging Infectious Diseases, University of Texas Medical Branch, Galveston, Texas, United States of America; U.S. Naval Medical Research Unit No. 2, Indonesia

## Abstract

**Background and purpose:**

The ability to track changes in gene expression following viral infection is paramount to understanding viral pathogenesis. This study was undertaken to evaluate the nCounter, a high throughput digital gene expression system, as a means to better understand West Nile virus (WNV) dissemination and the inflammatory response against WNV in the outbred Swiss Webster (SW) mouse model over the course of infection.

**Methodology:**

The nCounter Mouse Inflammation gene expression kit containing 179 inflammation related genes was used to analyze gene expression changes in multiple tissues over a nine day course of infection in SW mice following intraperitoneal injection with WNV. Protein expression levels for a subset of these cytokine/chemokine genes were determined using a multiplex protein detection system (BioPlex) and comparisons of protein/RNA expression levels made.

**Results:**

Expression analysis of spleen, lung, liver, kidney and brain of SW mice infected with WNV revealed that *Cxcl10* and *Il12b* are differentially expressed in all tissues tested except kidney. Data stratification of positively confirmed infected (WNV (+)) versus non-infected (WNV (−) tissues allowed differentiation of the systemic inflammatory gene response from tissue-specific responses arising from WNV infection. Significant (p<0.05) decrease in *C3ar1* was found in WNV (−) spleen. *Il23a* was significantly upregulated, while *Il10rb* was down-regulated in WNV (−) lung. *Il3* and *Mbl2* were down-regulated in WNV (−) liver. In WNV (+) livers, *Stat1*, *Tlr2*, chemokines *Cxcl1*, *Cxcl3*, *Cxcl9*, *Cxcl10*, cytokines *Il6*, *Il18*, cytokine-related gene *Il1r* and cytokine agonist *Ilrn* were significantly upregulated. In WNV (−) brain tissues, *Csf2* and *Cxcl10* were significantly upregulated. Similar gene and protein expression kinetics were found for *Ccl2*, *Ccl3*, *Ccl4 and Ccl5* and correlated with the presence of infectious virus. In summary, the utility of the nCounter platform for rapid identification of gene expression changes in SW mice associated with WNV infection was demonstrated.

## Author Summary

The nCounter, a high throughput digital molecular detection and quantitation system, was used to define the inflammatory response of SW mice to WNV infection following intraperitoneal injection in multiple tissues over a 9 day course of viral infection. Data analysis identified expression profiles common and unique to major tissues. Complementary protein suspension array analysis of a subset of genes demonstrated the expression kinetics of RNA and protein expression compared to infectious virus production. Data stratification of WNV (+) versus WNV (−) tissues allowed differentiation of the systemic inflammatory gene response versus tissue-specific responses due to infection with WNV over time. The results generated from this study will allow for further hypothesis generation and testing.

## Introduction

West Nile virus (WNV) is a neurotropic flavivirus endemic to parts of the Americas, Africa, Europe, the Middle East and Asia. WNV naturally cycles between mosquitoes and birds but also infects humans and other animals [1]. Humans infected with WNV develop a febrile illness that is self-resolving but a subset of cases, 20–25% will develop a febrile illness and 1 out of 150–250 WNV infections will progress to develop meningitis or encephalitis [2]–[4]. There are currently no approved antiviral therapies or vaccines for human use. Since its introduction to North America in 1999, WNV has become the leading cause of mosquito-borne epidemic encephalitis in the United States (US) and now poses a significant public health risk. From 1999 to 2012 the number of WNV infections has totaled 37,088 cases of which 16,114 cases were reported to be neuroinvasive and 1,549 were fatal (http://www.cdc.gov/ncidod/dvbid/westnile/index.htm). Since its introduction, the US has experienced annual epidemics of WNV disease that peaked in 2002–2003. Subsequently, the numbers of WNV cases remained relatively constant until a steady decline was observed beginning in 2008. However, 2012 saw a dramatic resurgence in the number of WNV cases reported [5] reinforcing the ongoing risk that WNV poses to human and animal health in North America and the unpredictable nature of outbreaks. Over the past few years, relatively large outbreaks of neuroinvasive disease in humans and horses involving multiple lineages of WNV have also been reported from several European countries [6]–[8]. Rapid screening technologies, coupled with a better understanding of the host response to viral infections would be of great value in responding to future outbreaks of such diseases.

Over the past decade, inbred mouse strains (i.e. C57BL/6, 129 Sv/Ev, BALB/C and C3H) of varying ages have been used extensively as model systems to study host responses and determinants of WNV neuroinvasive disease [9]–[11]. Using various inbred and knockout mouse strains, several laboratories have shown varying degrees of susceptibility to WNV infection, in addition to identifying aspects of innate and adaptive immune responses that offer protection against WNV infection [12]–[18]. Alternatively, ICR (CD-1) outbred mice have been used to demonstrate loss of neuroinvasiveness for some attenuated WNV strains. NIH Swiss or Swiss Webster outbred mice have been utilized to demonstrate differences in neuroinvasiveness and neurovirulence between naturally occurring and engineered WNV strains [19], to study molecular determinants of neuroinvasiveness in related lineage 1 WNV strains [20], and for evaluation of candidate vaccines and therapeutics [21]–[23].

In recent studies, microarray analysis has been used to detect changes in gene expression after viral infection to help better understand the immune mechanisms governing viral pathogenesis. Gene expression profiling of human embryonic kidney cells, human glioma cells and human retinal pigmented cells, as well as various mouse cell lines, infected with WNV *in vitro* have been reported [24]–[29]. With the development of multiple WNV mouse models, gene expression analysis has been applied to whole tissues of WNV-infected mice. The first microarray analysis of WNV mouse model used NIH Swiss mice and gene expression analysis focused primarily on brain, spleen and liver tissue [30]. A more recent study used *Mavs^−/−^*, *Ifnar^−/−^* or *Mavs^−/−^Ifnar^−/−^* double-knockout mice to demonstrate complex innate immune signaling regulated tissue tropism between the spleen and liver of WNV infected mice [31].

To better understand the dynamic changes in gene expression profiles across multiple peripheral tissues, high-throughput digital gene expression analysis was utilized to investigate the inflammatory response throughout the course of infection in the WNV Swiss Webster (SW) weanling mouse model. The nCounter molecular detection and quantitation system was used to examine how infection with highly neuroinvasive WNV strain 382–99 (often termed “NY99”) altered the kinetics of the inflammatory response through the course of infection in a common outbred mouse model of neuroinvasive WNV disease. The nCounter identifies and quantitates RNA transcripts free of enzymatic amplification, with high levels of sensitivity, linearity, and multiplexing, and can simultaneously analyze up to 850 individual transcripts at concentrations as low as 0.5fMs [32]. In this study, the utility of the nCounter system enabling rapid gene expression analysis for *in vivo* studies of WNV infection in mice was demonstrated. Genes common to multiple tissues that are differentially expressed when WNV RNA is detected and genes that are differentially expressed in a tissue-specific manner following active WNV infection across multiple tissues over the entire course of infection are reported.

## Materials and Methods

### Ethics Statement

All animal procedures complied with USDA guidelines and were conducted at the AAALAC-accredited Galveston National Laboratory ABSL-3 Laboratory at The University of Texas Medical Branch (UTMB; Galveston, TX) under protocol number 0508048A approved by the UTMB Institutional Animal Care and Use Committee.

### Cells and Viruses

West Nile virus (WNV) strain 382–99 was obtained from the World Reference Center for Emerging Viruses and Arboviruses (WRCEVA) at the University of Texas Medical Branch (UTMB). The virus was passaged once each in chicken embryo fibroblast cells (CE) and rhesus monkey kidney cells (LLC-MK2) prior to receipt at WRCEVA, and then twice in African green monkey kidney cells (Vero) to generate the stock used for this study. Vero E6 cells (ATCC, CRL1586) were maintained in MEM, supplemented with 10% heat-inactivated fetal bovine serum, 1,000 IU/ml penicillin, and 1,000 µg/ml streptomycin at 37°C/5% CO_2_.

### Viral Titers

WNV titers in culture supernatants, serum and homogenized tissue samples from animal experiments were determined by standard plaque assay on Vero cells. Briefly, samples were serially diluted in PBS in the range 1∶10^1^ to 1∶10^6^. One hundred microliters (100 µl) of sample was added to each well of a 12-well plate with a confluent monolayer of Vero cells. The plates were incubated for 30 minutes at room temperature, then 2 ml of overlay was added to each well (final concentration = 1% agar with 1× MEM, 2% Bovine Growth Serum, 0.6 mM L-glutamine, 1,000 IU/ml penicillin, 1,000 µg/ml streptomycin, and 1× MEM non-essential amino acids). Plates were incubated in a 37°C/5% CO_2_ incubator. At 2 days post-infection, 1 ml of overlay supplemented with 2% neutral red (Sigma) was added. Plaques were enumerated using a light box at day 3 post-infection.

### Mouse Experiments

A nine-day serial-sacrifice experiment was performed using thirty 3 to 4-week-old female Swiss Webster mice (Charles River Laboratories) injected intraperitoneally (i.p.) with 1,000 plaque-forming units (PFU) of WNV in a volume of 100 µl of PBS. Three mice were euthanized per day during days 1 to 9 post infection (p.i.). Three PBS mock infected control mice were sacrificed on day 1 p.i. Serum, brain, lung, liver, kidney, and spleen tissue were collected from each mouse. Each tissue sample was divided into two equivalent sized pieces. One piece was placed in 1 ml TRIzol (Life Technologies, Carlsbad CA) for RNA extraction. The other piece was placed in 500 µl PBS and homogenized for viral titer determination. Animal studies were performed at UTMB under ABSL3 conditions in accordance with a protocol approved by UTMB's Institutional Animal Care and Use Committee.

### Chemokine Analysis

Tissue homogenates were gamma-irradiated (5 megarads) prior to analysis at BSL2. Samples were processed according to manufacturer instructions and then analyzed using a Bio-Plex 200 system (Bio-Rad, Hercules, CA). Briefly, the tissue homogenates were centrifuged for 10 minutes at 1,000 rpm at 4°C to remove cellular debris. The cleared supernatants were aliquoted into 96-well plates in pre-determined wells; this plate was centrifuged at 1,250 rpm to remove any remaining debris. The supernatant was transferred to a 96-well flat bottom plate and processed for use on the Bio-Plex system. The cytokines were coupled to cytokine-specific multiplex beads (Bio-Rad) following the manufacturer's instructions using the pre-designed assays Bio-Plex Pro mouse cytokine 23-plex immunoassay. This panel measures the concentrations of cytokines and chemokines and includes interleukin *(IL)-1α/β*, *IL-2*, *IL-3*, *IL-4*, *IL-5*, *IL-6*, *IL-9*, *IL-10*, *IL-12 (p40, p70)*, *IL-13*, *IL-17*, eotaxin *(CCL11)*, *IFN-γ*, *KC (CXCL1)*, monocyte chemoattractant protein *(MCP)-1/(CCL2)*, granulocyte colony-stimulating factor (G-CSF/CSF3), granulocyte macrophage colony-stimulating factor (*GM-CSF/CSF2)*, macrophage inflammatory protein *(MIP)1α/β (CCL3/CCL4)*, tumor necrosis factor *(TNF)-α* and RANTES *(CCL5)*.

### Nucleic Acid Extractions

All sample RNAs were extracted from TRIzol (Life Technologies) treated brain, lung, kidney, liver and spleen tissue homogenates according to the manufacturer's instructions. One-fifth of the total volume of chloroform was added, mixed, incubated 15 min at 25°C and centrifuged at 5,000 rpm 15 min at 4°C. To the aqueous layer, a 70% volume of 100% isopropyl alcohol was added. Samples were mixed, incubated 10 min at 25°C then centrifuged at 13,000 rpm for 10 min at 4°C. The pellet was washed with 70% ethanol then centrifuged at 13,000 rpm for 10 min at 4°C, air dried briefly at room temperature (RT) and resuspended in RNase-free, DEPC treated water (Ambion, Austin, TX) and stored at −80°C until needed.

### qRT-PCR

All reactions were performed on 96 well FAST PCR plates (Applied Biosystems, Foster City, CA) in a total volume of 25 µl (20 µl master mix plus 5 µl sample) optimized for quantitative reverse transcriptase polymerase chain reaction (qRT-PCR). A volume of 20 µl qRT-PCR master mix was prepared per manufacturer instructions (AgPath-ID One-Step RT-PCR Kit, Life Technologies, Foster City, CA). Tissue homogenates were analyzed using 1 ng total RNA and reactions performed in triplicate. Each plate contained 3 negative (no template controls) and 3 positive controls containing 1,000 copies of Alien-armored RNA (Asuragen, Austin, TX). Alien armored RNA was prepared per manufacturer instructions and diluted to 200 copies/µl in water. Reactions were performed on ABI 7500 thermal cyclers (Life Technologies) under the following Real-Time Fast thermal cycling conditions: 45°C for 10 minutes for reverse transcription of cDNA synthesis, 95°C for 10 minutes for inactivation of the reverse transcriptase, activation of 25X RT-PCR Enzyme Mix, and denaturation of the RNA/cDNA hybrid; followed by amplification at 40 cycles of 97°C for 2 seconds and 60°C for 30 seconds. Oligonucleotide primers and probes were purchased from Biosearch Technologies, Inc. (Novato, CA). Primer and probe sequences were as follows; WNV 19-F 5′AGGTCCTTCGCAAGAGGT 3′, WNV 19-R 5′GYGCCAAGTGYACVACGT 3′, WNV 19-Probe 5′ FAM-GCCAAGATCAGCDTKCCAGCBA-BHQ1 3′. Upon receipt, oligos were reconstituted in sterile 1× Tris-EDTA Buffer (10 mM Tris-Cl, 1 mM EDTA, pH 8.0, Teknova, Hollister, CA) to a concentration of 100 mM. Working stocks were made by diluting primers and probes to a concentration of 10 mM with TE Buffer.

A standard curve of Cq vs. amount of infectious virus RNA added to each reaction was generated and used to determine the PFU equivalent viral RNA amounts reported using standard plaque assays on Vero cells and total RNA extracts from viral culture supernatants.

### nCounter Gene Expression Profiling

The nCounter Mouse Inflammation gene expression Kit was purchased from NanoString Technologies (Seattle, WA) and consists of 179 inflammation-related mouse genes (see Table S1) and 6 internal reference genes (http://www.nanostring.com/products/gene_expression_panels). The nCounter assay was performed using 100 ng of total RNA. Hybridization reactions were performed according to the manufacturer's instructions with 5 µl diluted sample preparation reaction and incubated at 65°C for a minimum of 18 h. Hybridized reactions were purified using the nCounter Prep Station (NanoString Technologies) and data collection was performed on the nCounter Digital Analyzer (NanoString Technologies) following the manufacturer's instructions. For each assay data collection was performed at maximum density (1155 fields of view). RNA concentrations were determined using a Qubit (*1.0*) Fluorometer (Life Technologies, Grand Island, NY).

The raw data were normalized to six genes within each tissue type with the lowest coefficient of variation using the nSolver software following manufacturer's instructions (NanoString Technologies). The normalized results are expressed as total mRNA counts.

### Statistical Analysis

Gene expression analysis was performed in Excel using a heteroscedastic Student's *t*-test and changes in gene expression profiles were consider significant if p<0.05; uncorrected for multiple T-test. For graphing purposes Prism version 6.0a (GraphPad Software, La Jolla, CA) was used. Correlation coefficient was measured using Pearson's *r*. To adjust for values equal 0 for PFU and genome equivalents, log (x+c) was used to normalize all PFU and genome values prior to statistical analysis; c = lowest measured value/2.

### Gene Association Analysis

The analysis used the raw Nanostring mouse gene expression data, captured as mRNA counts harvested from brain, liver, lung, spleen and kidney tissue. Each of the organ-specific datasets consisted of 179 distinct gene rows and 30 columns. The number of columns corresponded to 3 mice sacrificed each day for 9 days post-infection with WNV. An additional three columns corresponded to a single set of three “mock” values, harvested from un-infected mice to serve as a reference. The columns were further annotated by a ‘1’ if viral RNA was detected in the harvested tissue and ‘0’ if viral RNA was not detected. For each organ dataset, the data between ‘0’ and ‘1’ states was binned separately. Separate time series for ‘0’ and ‘1’ were generated by averaging over the ‘0’ and ‘1’ mice separately each day. Changes in gene expression between ‘0’ and ‘1’ states were then quantified. The expression level of a specific gene *y* was deemed to be up-regulated if the sign of median (*y*(1))-median(*y*(0)) was positive and down-regulated if the sign was negative. The magnitude of the change was quantified by calculating the Kullbeck-Leibler divergence [33] between the gene expression distributions in ‘0’ and ‘1’ states. Finally, a weighted gene expression correlation network was constructed by calculating the Pearson correlation coefficient between each pair of genes. The sign of the Pearson coefficient determined whether the time series were correlated (+) or anti-correlated(−).

### KEGG Pathway Enrichment and Protein-Protein Interaction Network Analysis

The Database for Annotation, Visualization and Integrated Discovery (DAVID) [34], [35] was used to perform KEGG functional enrichment of biological pathways significantly enriched with DEGs from individual tissues. A minimum count of 4 genes was used as the cut-off for determination of enriched biological pathways. The Benjamini-Hochberg correction was used to calculate the FDR of the p values; FDR<0.05.

Protein-protein interaction (PPI) networks were constructed using Search Tool for the Retrieval of Interacting Genes/Proteins (STRING) [36]. DEG list from individual tissues were used to construct PPI networks. PPI confidence networks were generated using the confidence view option at a medium confidence of 0.400. KEGG pathway annotation was visualized by using the KEGG enrichment function in STRING.

## Results

### WNV Infection in the Swiss Webster Mouse Model

**Figure 1:**
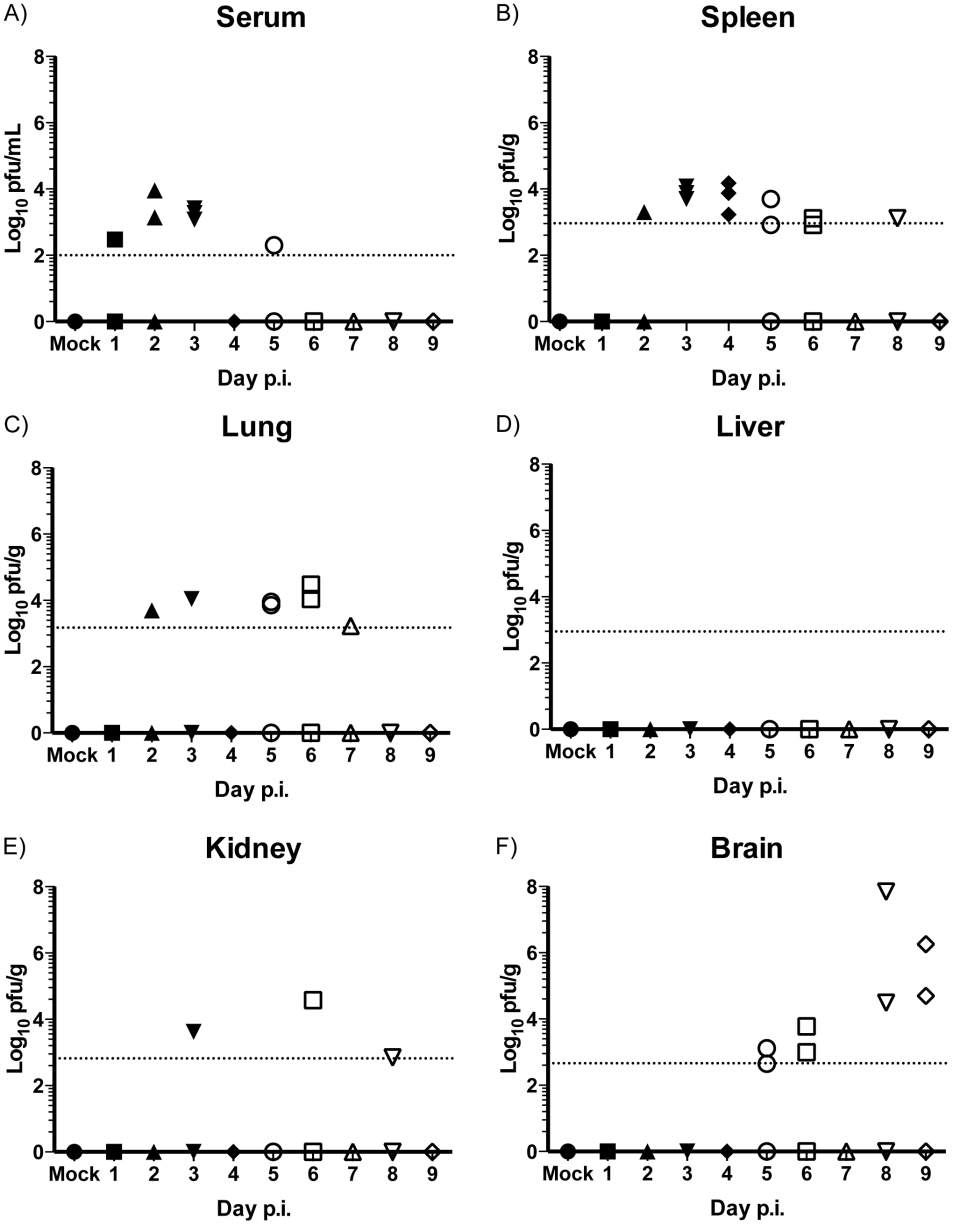
WNV viral titers in peripheral tissue of Swiss Webster outbred mice. Kinetics and levels of infectious WNV in (A) serum, (B) spleen, (C) lung, (D) liver, (E) kidney and (F) brain of infected SW mice determined by plaque assay. Data are presented as Log_10_ PFU per mL (serum) or per gram of tissue homogenate; *n* = 3 mice per time point. Dotted lines represent the limit of detection (LOD) of the assay, *n* = 3 mice per time point.

Outbred Swiss mice are highly susceptible to peripheral challenge with neuroinvasive strains of WNV [19], [23]. Despite these studies the WNV Swiss mouse model remains poorly characterized compared to inbred mouse models used to study WNV pathogenesis [12], [13]. To better characterize the WNV Swiss outbred mouse model, 3 to 4 week old Swiss Webster (SW) female mice were infected with the prototypical North American WNV NY99 (strain 382–99) via intraperitoneal (i.p.) injection. Three mice were randomly chosen and sacrificed daily during days 1 to 9 post infection (p.i.) and serum, spleen, lung, liver, kidney and brain were collected for determination of infectious WNV loads using a standard plaque assay and qRT-PCR protocol. Mice first displayed clinical signs of disease (hunching, ruffled fur, and tremors) on day 6 p.i. and one mouse was paralyzed on day 9 p.i. Consistent with previous studies in outbred and inbred mouse strains [12], [14], peak viremia (∼10^3.5^ PFU/mL) was detected 48–72 hours (h) p.i. and dropped below the plaque assay limit of detection (LOD) by day 6 p.i. (Figure 1A).

Dissemination of WNV to peripheral organs was detected by plaque assay as early as day 2 p.i. in the spleen and lungs (Figure 1B and 1C, respectively). In the spleen, the amount of infectious virus peaked at day 4 p.i. (∼10^4^ PFU/g) and was detectable up to day 8 p.i. Levels of infectious virus in the lung remained constant beginning on day 2 through day 7 p.i. (∼10^4^ PFU/g). No infectious virus was detected in the liver throughout the entire time course (Figure 1D). Infectious WNV was detected at day 3 p.i. in the kidney and was detectable up to day 8 p.i. (∼10^3.5^ PFU/g) (Figure 1E). Low levels of infectious virus (10^3^ PFU/g) were detected in the brain beginning at day 5 p.i. and with a marked increased at days 8 and 9 (>10^6^ PFU/g) (Figure 1 F). Increased viral titers correlated well with the onset of tremors at day 6 p.i.

**Figure 2:**
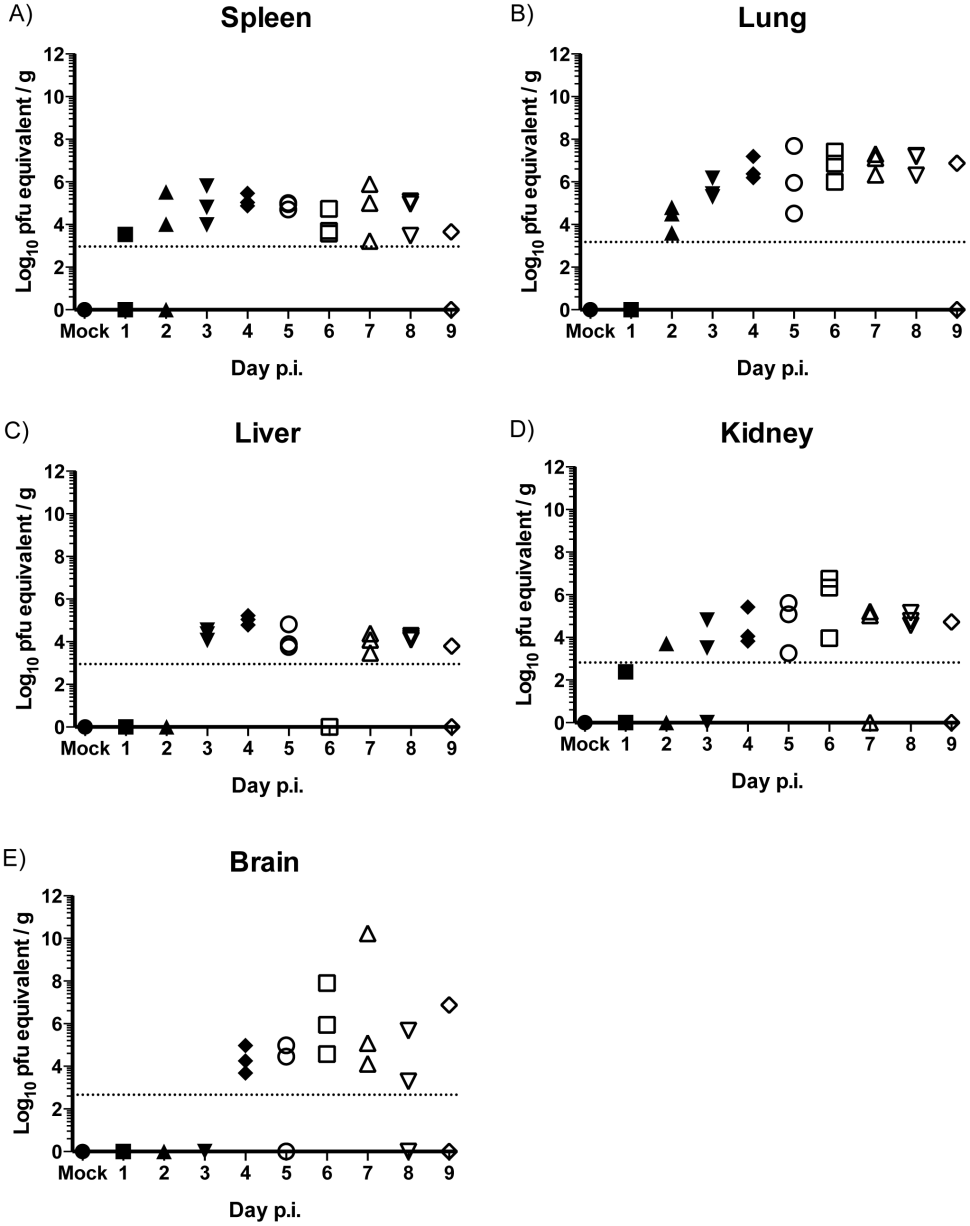
Levels of viral RNA in peripheral tissue of Swiss Webster outbred mice quantified by qRT-PCR. Total RNA was extracted from (A) spleen, (B) lung, (C) liver, (D) kidney and (E) brain of infected SW and levels of viral RNA determined. Data are presented as Log_10_ PFU equivalents per gram (g); Dotted lines are set at the same LOD as figure 1 for visual comparison of the two assays, *n* = 3 mice per time point.

Total RNA was extracted from brain, lung, liver, kidney and spleen homogenates for analysis using a WNV specific qRT-PCR protocol to determine infection by the presence or absence of genomic viral RNA. Using this method, viral RNA was detected on day 1 p.i. in the spleen (Figure 2A) and day 2 p.i. in the lung (Figure 2B). In contrast to the plaque assay, viral RNA was detected in the liver on day 3 p.i. (Figure 2C), on day 1 p.i. in the kidney (Figure 2D) and on day 4 p.i. in the brain (Figure 2E). The qRT-PCR method enabled determination of WNV RNA spread (and by extension, presence of virus) to visceral tissues with greater sensitivity than plaque-assay (summarized in Table 1). As with the infectious virus titers, at least some of the viral RNA detected in organ samples collected at days 1–3 may be attributed to presence of virus or virus-infected cells in blood. For the liver, viral RNA was detected only starting at day 3. Viral RNA “titer” in the liver from day 3 through day 9 was stable at 10^3^ to 10^5^ PFU equivalent/gram, potentially indicating that the viral RNA detected had a source other than infected liver tissue.

**Table 1:** WNV dissemination per tissue.

Tissue	plaque assay	qRT-PCR
**Spleen**	12/27	22/27
**Lung**	7/27	22/27
**Liver**	0/27	16/27
**Kidney**	3/27	19/27
**Brain**	8/27	14/27

Summary of WNV dissemination per tissue as determined by plaque assay and qRT-PCR.

### Dynamic Gene Expression Profile of Tissue-Specific Inflammatory Responses

The dynamic gene expression profiles of the inflammatory response in WNV-infected SW outbred mice were analyzed using the nCounter system, a next generation digital gene expression system that allows for multiplex-gene expression analysis in one reaction without the need of enzymatic RNA amplification. A predefined 179-gene expression mouse inflammation (Table S1) panel was used to analyze daily changes in gene expression profiles (GEP) in the spleen, lung, liver, kidney and brain in mice infected with WNV compared to mock control SW mice. A general picture of GEP trends was obtained by comparing significance versus fold changes for each gene per day. This preliminary analysis identified 41 differentially expressed genes (DEG) in the spleen, 114 in the lung, 19 in the kidney, 61 in the liver and 9 in the brain (Figure S1). The very large number of DEG identified in the lung from 4 to 8 days p.i. was an unexpected finding and to our knowledge no previous studies have shown this.

**Figure 3:**
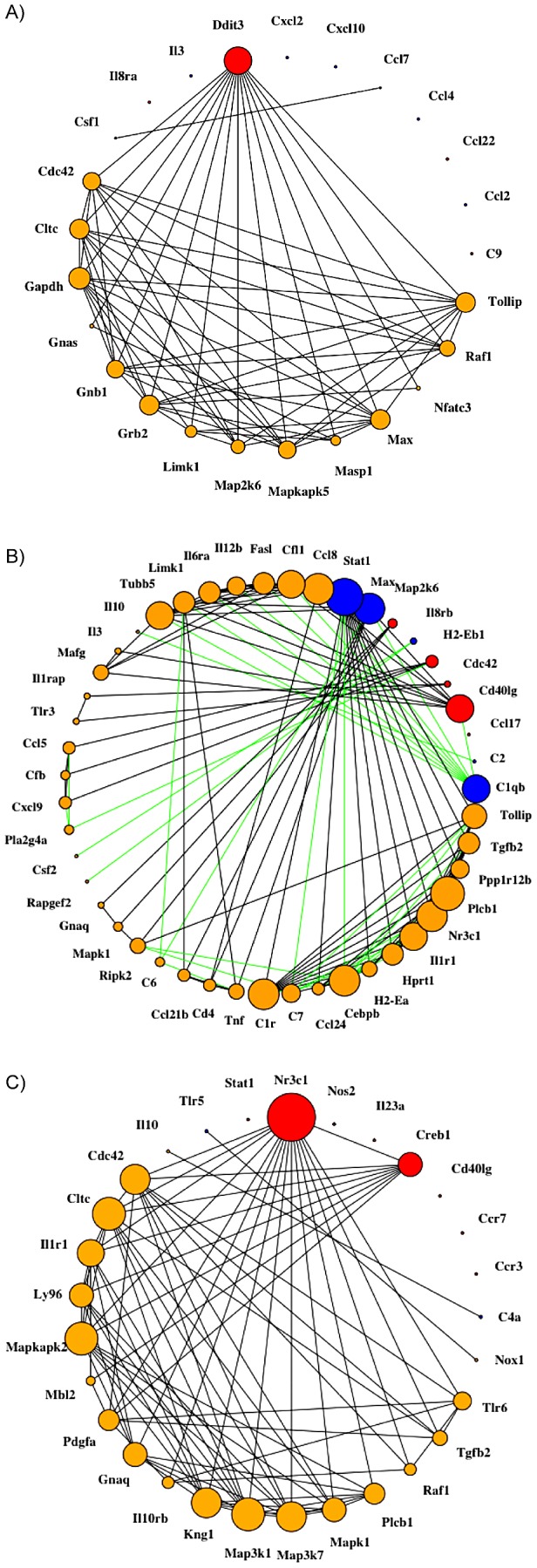
Maximally transition-relevant gene expression networks for (A) brain, (B) spleen, and (C) kidney in WNV-infected Swiss Webster mice. Blue and red nodes are *maximally transition-relevant* (MTR) genes, defined as those genes that changed their expression levels above the 95th percentile of the tissue-specific K-L divergence calculated between gene expression in WNV RNA(+) infected tissue relative to WNV RNA(−) infected tissue. Blue indicates upregulation of the gene and red labels down-regulation. Edges between two genes X and Y in the networks indicate a Pearson correlation coefficient P(X,Y) with an absolute value over 97.5-th percentile. Orange nodes are first-degree neighbors of the MTR genes. Black edges indicate a positive correlation coefficient (i.e. between the two genesand green indicates a negative coefficient (anti-correlation). Nodes are sized by degree, i.e. how many other genes the node is connected to. Lung is not shown because there were no connections present at the significance threshold used. Liver is not shown as there was no conclusive evidence of liver infection.

Genes which had changes in their expression levels most strongly correlated with detection of viral RNA were identified (Red and Blue circles, Figure 3) by generating gene expression networks for brain, liver, spleen, and kidney. This analysis identified genes that changed their expression levels above the 95th percentile of the tissue-specific Kullbeck-Leibler (K-L) divergence [33] calculated between gene expression in WNV RNA(+) tissue relative to WNV RNA(−) tissue. This general analysis revealed that expression of DNA damage inducible transcript 3 (DDIT3), an apoptosis promoter gene was down-regulated in infected brain tissue and was associated with a reduction in expression of 14 of other genes in that tissue. For spleen, *Stat1*, *Max*, and *C1qb*, were all upregulated in infected tissue and showing an association with a large number of other genes in that tissue (large blue circles). *Cd401g* was down-regulated in spleen and also showed association with a large number of other genes in this tissue (large red circle). For kidney, two transcriptional effectors, *Nr3c1* and *Creb1* were the genes showing an association with a large number of other genes. Even though the lung showed 114 DEG, no connections between genes were found at the significance threshold used in this analysis. Liver analysis is not shown as we could not conclusively demonstrate liver infection thus rendering correlations non-informative.

**Figure 4:**
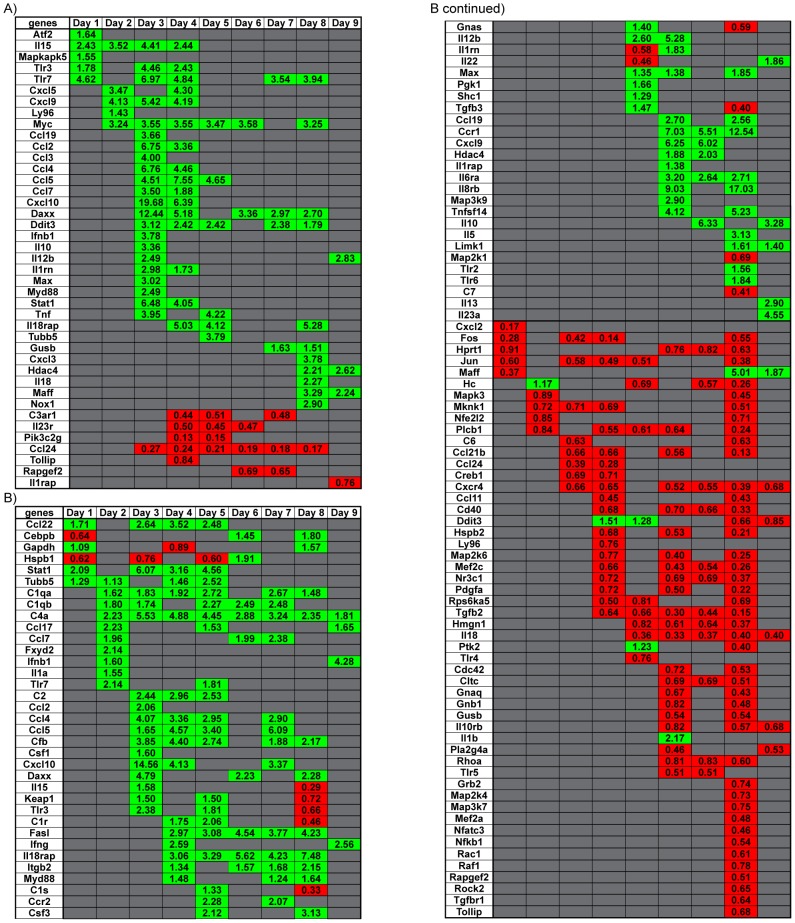
Dynamic Gene Expression profiles in various tissues over course of infection. Heatmap of specific genes in (A) spleen, and (B) lung that had a significant change in gene expression per day over the course of infection. Green shaded boxes represent genes that were upregulated and red shaded boxes represent genes that were downregulated. Fold change for each gene is denoted within each shaded box. Data points represent n = 3 mice per day, p<0.05 consider statistically significant.

**Figure 5:**
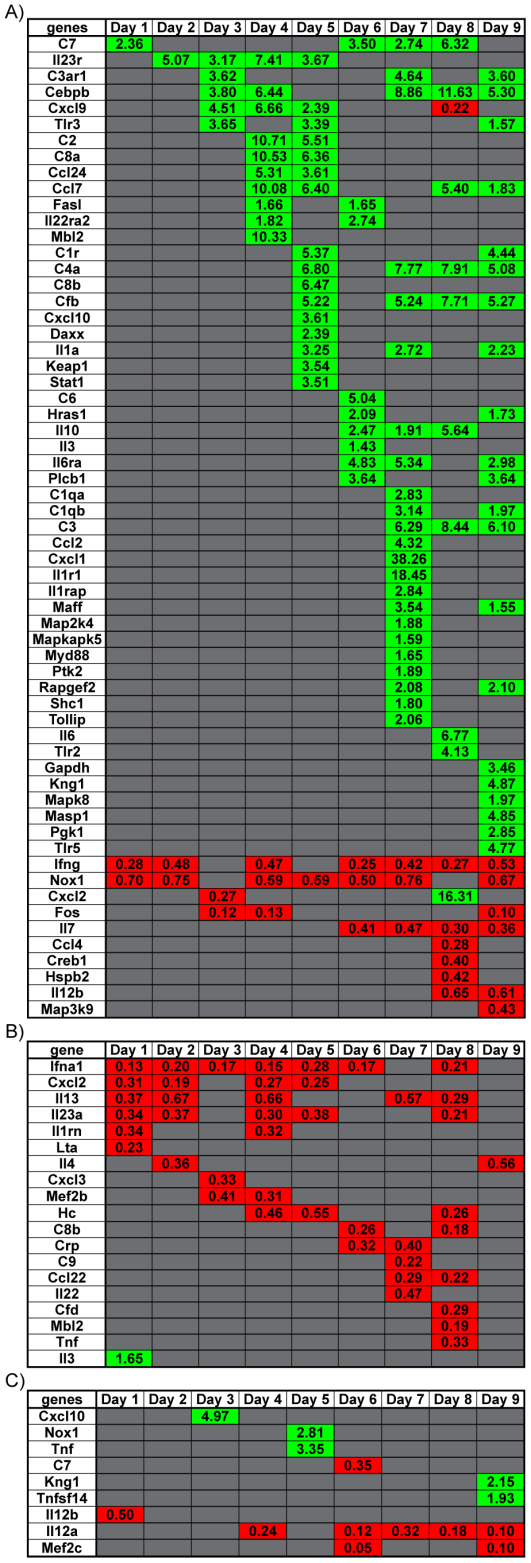
Dynamic Gene Expression profiles in various tissues over course of infection. Heatmap of specific genes in (A) liver, (B) kidney, and (C) brain that had a significant change in gene expression per day over the course of infection. Green shaded boxes represent genes that were upregulated and red shaded boxes represent genes that were downregulated. Fold change for each gene is denoted within each shaded box. Data points represent n = 3 mice per day, p<0.05 consider statistically significant.

Gene clustering revealed the majority of the DEG in the spleen was observed on days 3 to 5 p.i. (Figure 4A) coinciding with observed peak viral titers in the spleen (Figure 1B). In the lung, the number of up-regulated genes roughly equaled that of down-regulated genes between days 5 to 8 p.i. (Figure 4B) around the time peak viral titers were observed (Figure 1C). No infectious virus was detectable in the liver. However, the number of DEG that were upregulated between days 4 to 9 p.i. (Figure 5A) followed similar trends when compared to observed genomic WNV RNA detected (Figure 2C). In the kidney, the number of DEG peaks at day 8 p.i. (Figure 5B), although infectious virus was not consistently detected and viral RNA peaked at day 6 (Figure 2D). Interestingly, after day 1 p.i., all DEG in the kidney were down-regulated. In the brain, the number of DEG was small compared to the spleen and lung and these were mostly down regulated (Figure 5C).

### Differentially Expressed Genes Common or Uniquely Expressed Across Multiple Tissues

Using the nCounter system, changes in gene expression profiles were tracked in multiple tissues through an entire course of infection and genes that are commonly and uniquely differentially expressed in multiple tissues were identified. To our knowledge, such a comprehensive analysis of the inflammatory response to viral infection in an outbred animal model has not been previously described or attempted through other methods.

Chemokine gene *Cxcl10* and the cytokine gene *Il12b* were differentially expressed in four of five tissues (Figures 4 & 5 and Table 2). Consistent with previous reports, *Cxcl10* was highly expressed in the brain [37]–[39], spleen [37], liver [31] and lung (described here). The expression of *Il12b* has been reported to decrease in the blood and brain of *Tlr7^−/−^* mice infected with WNV [40]. This study also showed *Il12b* expression levels lowered in the brain and liver, while *Il12b* expression levels were higher in the spleen and lung.

**Table 2:** Common and uniquely differentially expressed genes in tissues from WNV infected SW mice.

Distribution	Tissues	Number of Genes	Gene Function					
			Chemokine Ligands & Receptors	Cytokine Lignads & Receptors	Complement	Signal Transduction	Toll-like Receptors and Associated Proteins	Transcriptional Regulators
Common in 4 tissues	Spleen/Lung/Liver/Brain	2	*Cxcl10*	*Il12b*				
Common in 3 tissues	Spleen/Lung/Liver	14	*Ccl2 Ccl4 Ccl7 Ccl24 Cxcl9*	*Il1rap Il10*		*Myd88 Rapgef2 Daxx*	*Tollip Tlr3*	*Maff Stat1*
	Spleen/Lung/Kidney	1	*Il1rn*					
	Spleen/Liver/Brain	1				*Nox1*		
	Spleen/Kidney/Brain	1		*Tnf*				
	Lung/Liver/Brain	1	*Cxcl2*					
	Lung/Liver/Brain	1			*C7*			
Common in 2 tissues	Spleen/Lung	13	*Ccl5 Ccl19*	*Il15 Il18 Il18rap Ifnb1*		*Gusb Tubb5*	*Tlr7 Ly96*	*Ddit3 Hdac4 Max*
	Spleen/Liver	3	*C3ar1*	*Il23r*		*Mapkapk5*		
	Spleen/Kidney	1	*Cxcl3*					
	Lung/Liver	25		*Il1a Il6ra Ifng*	*C1qa C1qb C1r C2 C4a C6 Cfb*	*Fasl Fos Gapdh Hspb2 Map3k9 Map2k4 Pgk1 Plcb1 Ptk2 Shc1*	*Tlr2 Tlr5*	*Cebpb Creb1 Keap1*
	Lung/Kidney	5	*Ccl22*	*Il13 Il22 Il23a*	*Hc*			
	Lung/Brain	2		*Tnfsf14*				*Mef2c*
	Liver/Kidney	3		*Il3*	*C8b Mbl2*			
	Liver/Brain	1	*Kng1*					
Tissue-Specific	Spleen	5	*Ccl3 Cxcl5*			*Pik3c2g*		*Atf2 Myc*
	Lung	48	*Ccl11 Ccl17 Ccl21b Ccr1 Ccr2 Csf1 Csf3 Cxcr4*	*Il1b Il10rb Il5 Il8rb*		*Cd40 Cdc42 Cltc Fxyd2 Gnas Gnaq Gnb1 Grb2 Hprt1 Hspb1 Itgb2 Limk1 Map2k1 Map2k6 Mapk3 Map3k7 Mknk1 Nfkb1 Pdgfa Pla2g4a Rac1 Raf1 Rhoa Rps6ka5 Rock2 Tgfb2 Tgfb3 Tgfbr1*	*Tlr4 Tlr6*	*Hmgn Jun Mef2a Nfatc3 Nfe2l2 Nr3c1*
	Liver	10	*Cxcl1*	*Il1r1 Il22ra2 Il6 Il7*	*C3 C8a Masp1*	*Hras1 Mapk8*		
	Kidney	7		*Il4 Ifna1 Lta*	*C9 Cfd Crp*			*Mef2b*
	Brain	1		*Il12a*				

Commonly and uniquely expressed genes that were differentially expressed in spleen, lung liver, kidney and brain. All changes were found to be statistically significant, p<0.05.

DEG in three of the five tissues collected were then analyzed. DEG common to the spleen, lung and liver totaled 14 and included seven chemokine and cytokine genes, 3 signal transduction genes, 2 TLR genes and 2 transcriptional regulators (summarized in Table 2). DEG common to the lung, liver and kidney were alternative complement pathway gene *Cfb*, and chemokine gene *Cxcl2*. A complement factor gene (C7) is differentially expressed (DE) in the lung, liver and brain. A signal transduction gene (*Nox1*) is DE in the spleen, liver and brain. A cytokine gene (*Tnf*) is DE in the spleen, kidney and brain (Table 2). Large numbers of DEG were found to be common to two tissue types and a number of genes were uniquely expressed in a tissue-specific manner (summarized in Table 2).

In order to better understand biological mechanisms that may be involved in the inflammatory response against WNV infection, the list of DEG from each individual tissue was then used to perform a KEGG pathway enrichment analysis using DAVID [34], [35]. This analysis identified several biological pathways that were consistently activated in the tissues studied (Table 3). Among the top pathways identified in most tissues were cytokine-cytokine receptor interactions, Toll-like receptor signaling, chemokine signaling, JAK-STAT signaling, MAPK signaling and complement and coagulation cascades. The complete pathway enrichment for each tissue is summarized in (Table 3). Brain was not included in this analysis due to an insufficient number of DEG identified.

**Table 3:** KEGG pathway enrichment from tissue-specific, differentially expressed genes.

Spleen					
Term	Genes	Count	%	p Value	Benjamini
Cytokine-cytokine receptor interaction	CCL3, IL18RAP, TNF, CCL2, IL23R, CXCL5, IL18, CXCL9, CCL19, IL15, CCL5, CCL4, IL10, CCL7, CXCL10, CCL24, IFNB1, IL1RAP, IL12B	19	46.3	4.90E-16	2.31E-14
Toll-like receptor signaling pathway	CCL3, TNF, TOLLIP, LY96, CXCL9, TLR3, CCL5, STAT1, TLR7, CCL4, CXCL10, MYD88, IFNB1, IL12B	14	34.1	8.93E-15	2.31E-13
Chemokine signaling pathway	CCL24, CCL3, CCL2, CXCL5, CXCL9, CCL19, CCL5, STAT1, CCL4, CCL7, CXCL10	11	26.8	1.00E-07	1.73E-06
Jak-STAT signaling pathway	IL23R, IFNB1, IL12B, IL15, STAT1, MYC, IL10	7	17.1	3.16E-04	4.10E-03
Cytosolic DNA-sensing pathway	IFNB1, IL18, CCL5, CCL4, CXCL10	5	12.2	3.54E-04	3.68E-03
NOD-like receptor signaling pathway	TNF, CCL2, IL18, CCL5, CCL7	5	12.2	5.62E-04	4.86E-03
MAPK signaling pathway	MAX, TNF, MAPKAPK5, RAPGEF2, DAXX, MYC, DDIT3, ATF2	8	19.5	1.09E-03	8.07E-03
*RIG-I-like receptor signaling pathway	TNF, IFNB1, IL12B, CXCL10	4	9.8	8.60E-03	5.46E-02

Lung					
Term	Genes	Count	%	p Value	Benjamini
Cytokine-cytokine receptor interaction	CSF3, CCL2, PDGFA, CCR1, CSF1, IL18, CXCL2, TGFB3, CXCL9, IL13, TNFSF14, IL15, CCL5, CCL4, CCL7, IL10, TGFB2, CXCL10, CCL24, CCL22, IL23A, CXCR4, IL10RB, IFNG, IL1RAP, IL1B, FASL, IL1A, IL18RAP, IL5, TGFBR1, CCL19, CCL21B, CD40, IL22, IL6RA, CCL17, CCL11, IFNB1, CCR2, IL12B	41	37.3	8.27E-30	7.86E-28
Toll-like receptor signaling pathway	TOLLIP, TLR2, CXCL9, TLR3, TLR4, NFKB1, TLR5, CCL5, TLR6, CCL4, TLR7, CXCL10, MAP3K7, FOS, MYD88, RAC1, IL1B, MAP2K6, MAP2K1, LY96, MAP2K4, CD40, STAT1, IFNB1, JUN, MAPK3, IL12B	27	24.5	1.11E-24	5.27E-23
Chemokine signaling pathway	CCL2, GRB2, CCR1, CXCL2, CXCL9, NFKB1, CCL5, CCL4, CCL7, CXCL10, CCL24, CDC42, CCL22, PTK2, CXCR4, RAC1, RHOA, SHC1, PLCB1, MAP2K1, ROCK2, RAF1, CCL19, CCL21B, STAT1, CCL17, CCL11, GNB1, CCR2, MAPK3	30	27.3	6.25E-21	1.98E-19
MAPK signaling pathway	MEF2C, GRB2, PDGFA, TGFB3, MKNK1, NFKB1, DAXX, TGFB2, MAP3K7, FOS, CDC42, MAX, RAC1, IL1B, FASL, RAPGEF2, IL1A, MAP2K6, MAP2K1, TGFBR1, MAP2K4, RAF1, DDIT3, RPS6KA5, PLA2G4A, JUN, MAPK3, HSPB1	28	25.5	2.24E-14	5.30E-13
T cell receptor signaling pathway	IL5, MAP2K1, GRB2, RAF1, NFKB1, IL10, MAP3K7, CDC42, FOS, JUN, IFNG, MAPK3, RHOA, NFATC3	14	12.7	9.54E-08	1.51E-06
NOD-like receptor signaling pathway	CCL11, MAP3K7, CCL2, IL18, CXCL2, MAPK3, IL1B, NFKB1, CCL5, CCL7	10	9.1	9.74E-07	1.16E-05
Jak-STAT signaling pathway	CSF3, IL5, GRB2, IL13, IL15, STAT1, IL22, IL10, IL6RA, IL23A, IL10RB, IFNB1, IFNG, IL12B	14	12.7	1.84E-06	1.95E-05
Complement and coagulation cascades	C1QA, C1QB, C7, C4A, HC, CFB, C6, C1S, C2	9	8.2	4.14E-05	2.31E-04
Natural killer cell mediated cytotoxicity	MAP2K1, GRB2, IFNB1, IFNG, RAC1, MAPK3, RAF1, SHC1, ITGB2, FASL, NFATC3	11	10.0	4.49E-05	2.37E-04
VEGF signaling pathway	CDC42, PLA2G4A, PTK2, MAP2K1, RAC1, MAPK3, RAF1, HSPB1, NFATC3	9	8.2	4.56E-05	2.28E-04
B cell receptor signaling pathway	FOS, MAP2K1, GRB2, JUN, RAC1, MAPK3, RAF1, NFKB1, NFATC3	9	8.2	6.62E-05	3.14E-04
Gap junction	MAP2K1, GNAQ, PDGFA, GRB2, MAPK3, TUBB5, RAF1, GNAS, PLCB1	9	8.2	1.11E-04	5.03E-04
Intestinal immune network for IgA production	IL5, CXCR4, TGFB3, IL15, CD40, IL10, TGFB2	7	6.4	3.16E-04	1.37E-03
Cytosolic DNA-sensing pathway	IFNB1, IL18, IL1B, NFKB1, CCL5, CCL4, CXCL10	7	6.4	3.50E-04	1.45E-03
Focal adhesion	CDC42, PTK2, MAP2K1, ROCK2, PDGFA, GRB2, JUN, RAC1, MAPK3, RHOA, RAF1, SHC1	12	10.9	6.02E-04	2.38E-03
TGF-beta signaling pathway	ROCK2, TGFBR1, IFNG, MAPK3, TGFB3, RHOA, TGFB2	7	6.4	3.90E-03	1.19E-02
Regulation of actin cytoskeleton	CDC42, PTK2, MAP2K1, ROCK2, PDGFA, LIMK1, RAC1, MAPK3, RHOA, RAF1, ITGB2	11	10.0	4.29E-03	1.27E-02
Fc gamma R-mediated phagocytosis	CDC42, PLA2G4A, MAP2K1, LIMK1, RAC1, MAPK3, RAF1	7	6.4	6.97E-03	1.88E-02
Adherens junction	MAP3K7, CDC42, TGFBR1, RAC1, MAPK3, RHOA	6	5.5	1.01E-02	2.58E-02
Hematopoietic cell lineage	CSF3, IL5, CSF1, IL1B, IL1A, IL6RA	6	5.5	1.52E-02	3.75E-02
*RIG-I-like receptor signaling pathway	MAP3K7, IFNB1, NFKB1, IL12B, CXCL10	5	4.5	3.02E-02	6.56E-02

Liver					
Term	Genes	Count	%	p Value	Benjamini
Cytokine-cytokine receptor interaction	CXCL1, IL3, IL6, IL1R1, IL23R, CCL2, IL7, CXCL2, CXCL9, CCL4, IL10, CCL7, CXCL10, IL6RA, CCL24, IFNG, IL1RAP, FASL, IL12B, IL1A, IL22RA2	21	35.0	2.31E-14	1.64E-12
Complement and coagulation cascades	KNG1, MBL2, C3AR1, C7, MASP1, C4A, CFB, C3, C6, C1QA, C8A, C8B, C1QB, C2	14	23.3	1.04E-13	3.68E-12
Toll-like receptor signaling pathway	IL6, TOLLIP, MAP2K4, CXCL9, TLR2, TLR3, TLR5, STAT1, CCL4, CXCL10, FOS, MYD88, MAPK8, IL12B	14	23.3	4.29E-12	1.02E-10
Chemokine signaling pathway	CXCL1, CCL2, CXCL2, CXCL9, STAT1, CCL4, CCL7, CXCL10, HRAS1, CCL24, PTK2, SHC1, PLCB1	13	21.7	9.62E-08	1.14E-06
Jak-STAT signaling pathway	IL3, IL6, IL23R, IL7, IFNG, IL12B, STAT1, IL10, IL6RA, IL22RA2	10	16.7	1.14E-05	1.15E-04
NOD-like receptor signaling pathway	CXCL1, IL6, CCL2, CXCL2, MAPK8, CCL7	6	10.0	2.87E-04	2.55E-03
MAPK signaling pathway	FOS, IL1R1, MAPKAPK5, MAP2K4, MAPK8, FASL, RAPGEF2, DAXX, IL1A, HRAS1	10	16.7	8.18E-04	6.43E-03
Hematopoietic cell lineage	IL3, IL1R1, IL6, IL7, IL1A, IL6RA	6	10.0	1.17E-03	8.27E-03
Apoptosis	IL3, IL1R1, MYD88, IL1RAP, FASL, IL1A	6	10.0	1.37E-03	8.80E-03

Kidney					
Term	Genes	Count	%	p Value	Benjamini
Cytokine-cytokine receptor interaction	IL4, IL3, CCL22, IFNA1, TNF, IL23A, CXCL2, IL13, IL22, LTA	10	52.6	6.54E-10	2.03E-08
Jak-STAT signaling pathway	IL4, IL3, IFNA1, IL23A, IL13, IL22	6	31.6	2.02E-05	3.12E-04
Complement and coagulation cascades	MBL2, C8B, C9, HC, CFD	5	26.3	2.44E-05	2.52E-04

KEGG pathway enrichment from tissue specific DEGs: FDR<0.05.

* Indicates Biological pathway greater than the set FDR but is included, as this pathway has been implicated in WNV infections.

STRING (Search Tool for the Retrieval of Interacting Genes/Proteins) was used to identify protein-protein interactions (PPI) networks for all DEG within each tissue [36]. STRING is a database of known and predicted functions that are derived from direct (physical) and indirect (functional) associations derived from genomic context, high throughput experiments, conserved coexpression and previous knowledge [36]. Networks for spleen, kidney, lung, liver and brain were generated (shown in Figure S2A–E). Brain was included in this analysis even though no biological pathways were identified for this organ using DAVID as a PPI network could be generated using STRING with the limited number of DEG. Consistent with the analysis of DEGs described above, the most significantly activated pathways within the PPI network for each tissue (with the exception of the brain) were cytokine-cytokine receptor interactions for the lung (Figure S3), liver (Figure S4), spleen (Figure S5) and kidney (Figure S6), followed by Toll-like receptor signaling pathways for the lung (Figure S7) and spleen (Figure S8), complement and coagulation cascades for the liver (Figure S9) and JAK-STAT signaling pathway for the kidney (Figure S10). High throughput gene expression analysis enabled identification of common and differentially expressed genes in various tissues and a pathway enrichment analysis that allowed for the identification and visualization of relevant pathways that were enriched with DEG to better understand biological processes involved in WNV of the SW mouse model.

### Expression of Inflammatory Response Genes and Their Correlation to Infectious WNV Titers

Having determined DEG in the various tissues analyzed, correlations between amounts of virus and/or viral RNA (as determined by viral titers and/or viral RNA via qRT-PCR) and DEG identified were sought. Pearson correlation was performed with cutoff for significance at p<0.05.

**Figure 6:**
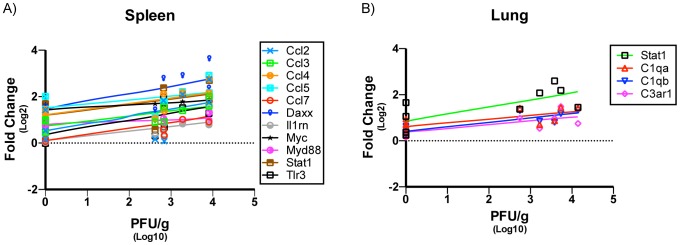
Correlation of inflammatory response genes to WNV viral titers in spleen and lung. Following gene expression analysis a Pearson's correlation test was performed on all genes that were determined to be significant. Gene expression versus viral titers (PFU/g) correlation was determined to be significant if p<0.05. A positive linear dependence was found for several genes in the A) Spleen and B) Lung of WNV infected SW mice; n = 3 mice per day.

Significant correlation was observed between virus titers and DEG in the spleen and lung (Figure 6 A and B) but not the kidney and brain (data not shown). For example, in the spleen *Ccl2*, *Ccl3*, *Ccl4*, *Ccl5*, *Daxx*, *Il1rn*, *Myc*, *Myd88*, *Stat1* and *Tlr3* were shown to have a significant positive correlation with WNV titers (Figure 6A), while genes that had a significant positive correlation with virus titers in the lung were *Stat1*, *C1qa*, *C1qb and C3ar1* (Figure 6B).

**Figure 7:**
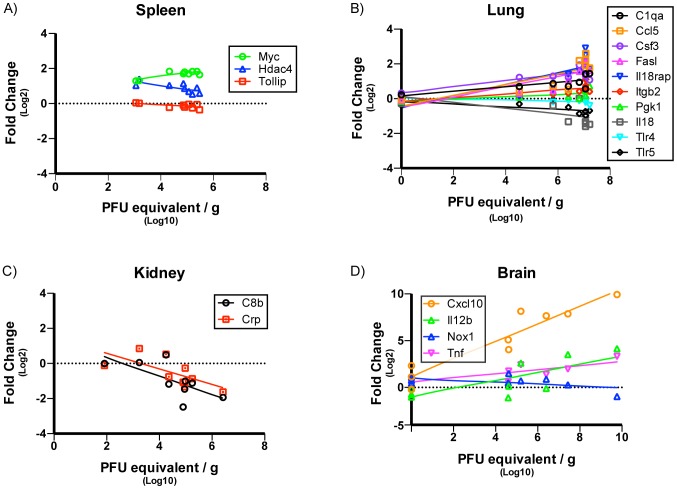
Correlation of inflammatory response genes to WNV PFU equivalents in spleen, lung, kidney and Brain. A Pearson's correlation test was performed as previously described. Gene expression versus PFU equivalents (PFU/g) correlation was determined to be significant if p<0.05. Both positive and negative linear dependence were found for several genes in the (A) spleen, (B) lung and (C) kidney and (D) brain of WNV infected SW mice; n = 3 mice per day.

Significant correlation was detected between amount of viral RNA present (expressed as PFU_equivalents/g_) and DEG in the spleen, lung, kidney and brain (Figure 7). No such correlations could be made for any genes in the liver. A positive correlation for *Myc* and negative correlations for *Hdac4* and *Tollip* were observed in the spleen (Figure 7A). In the lung, positive correlations were found for *C1qa*, *Ccl5*, *Csf3*, *Il18rap*, *Itgb2*, *Fasl* and *Pgk1* and negative correlations were found for *Il18*, *Tlr4* and *Tlr5* (Figure 7B). In the kidney there were negative correlations for *C8b* and *Crp* (Figure 7C). Positive correlations for *Cxcl10*, *Il12b* and *Tnf* were found in the brain while a negative correlation was found for *Nox1* in that tissue (Figure 7D). Overall the analysis performed here has allowed correlation of gene expression profiles using two parameters (i.e. infectious virus and genomic RNA titers) commonly used to quantitate WNV infections. Whether these host gene changes are correlates of protection or disease in the individual tissues remains to be investigated in future studies.

### Protein Expression and Viral Kinetics in Tissue-Specific Responses

**Figure 8:**
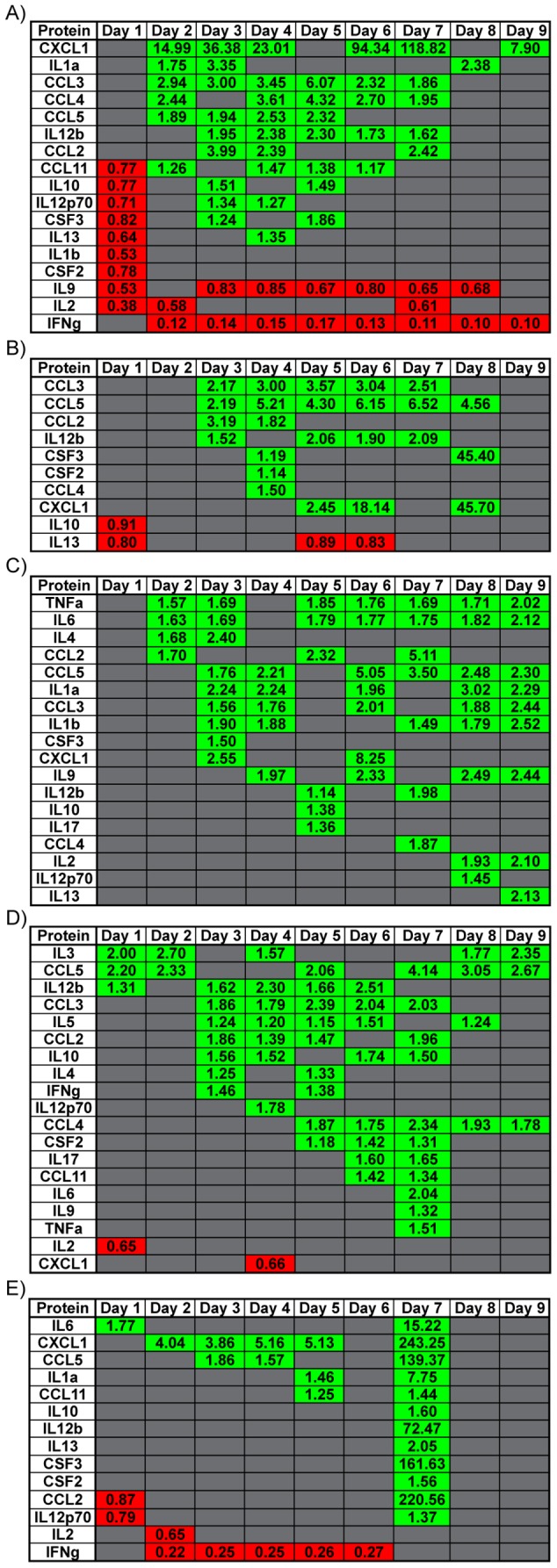
Dynamic Protein Expression profiles in various tissues over course of infection. Heatmap of specific genes in (A) spleen, (B) lung, (C) liver, (D) kidney and (E) brain that had a significant change in protein expression per day over the course of infection. Green shaded boxes represent genes that were upregulated and red shaded boxes represent genes that were downregulated. Fold change for each gene is denoted within each shaded box. Data points represent n = 3 mice per day, p<0.05 consider statistically significant.

Expression levels for 23 cytokine proteins in homogenized tissue extracts (constituting a subset of the 179 genes analyzed by the nCounter) were determined using a multiplex immunoassay (Bio-Plex Pro). Changes in protein expression, measured as mean fluorescence intensity (MFI), were analyzed using a heteroscedastic student's t-test and changes in protein expression were considered significant for p<0.05. A general picture of protein expression trends was obtained by comparing significance versus fold changes for each protein MFI per day on a volcano plot for spleen, lung, liver, kidney and brain (data not shown). These analyses identified 19 cytokine proteins in the kidney, 18 in the liver, 17 in spleen, 14 in the brain and 10 in the lung that were differentially expressed (data not shown). In the spleen, multiple proteins were down-regulated at day 1 p.i. however, the majority of proteins were significantly upregulated at later time points and DE proteins peaked between days 3 and 4 p.i. (Figure 8A). In the lung and kidney, the numbers of DE proteins were mostly upregulated and peaked at day 4 p.i. for the lung and day 7 p.i. for the kidney (Figures 8B and 8D). In the liver, all significantly DE proteins were upregulated with maximal numbers of such DE proteins observed on days 3, 8 and 9 (Figure 8C). In contrast to the other tissues where a number of proteins were DE over multiple days, in the brain DE proteins were concentrated at day 7 (Figure 8E).

### Viral Infection and Chemokine Expression Kinetics

**Figure 9:**
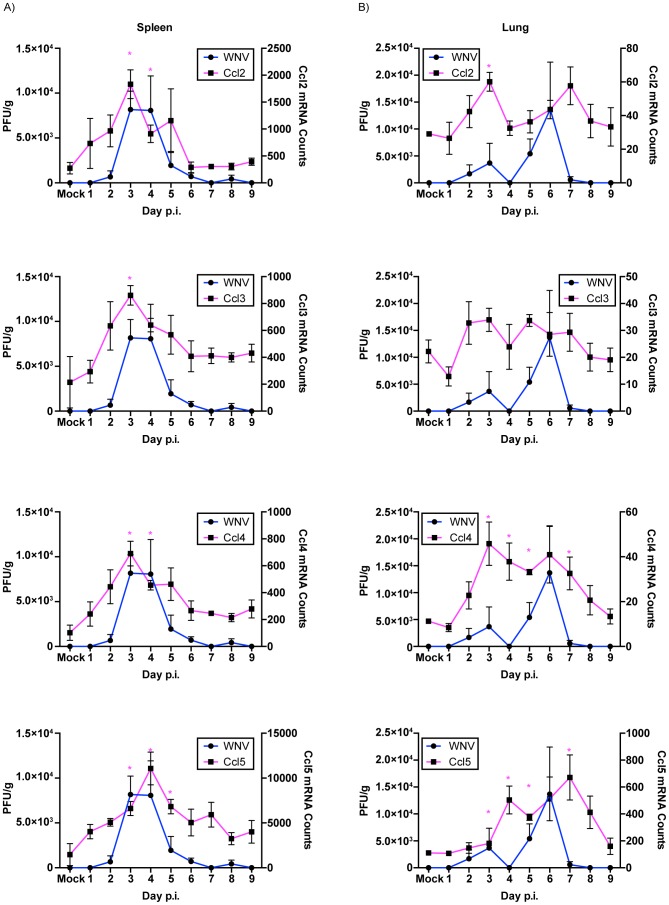
Kinetics of gene expression and WNV viral titers in the (A) spleen and (B) lung of WNV infected SW mice. WNV titers (left Y-axis) and gene expression levels (right Y-Axis) were plotted over time and the kinetics of viral titers and gene expression analyzed. *CCL2*, *CCL3*, *CCL4* and *CCL5* had similar expression kinetics that mirrored those of WNV titers in the tissues. Error bars represent SEM; n = 3 mice per day. * represents statistically significant changes for mRNA (magenta).

**Figure 10:**
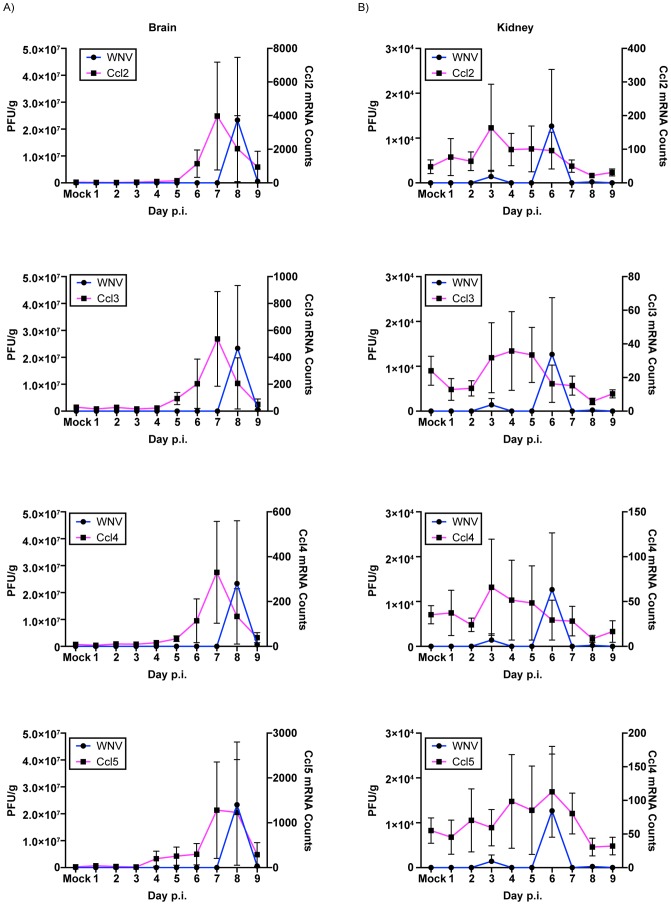
Kinetics of gene expression and WNV viral titers in the (A) kidney and (B) brain of WNV infected SW mice. WNV titers (left Y-axis) and gene expression levels (right Y-Axis) were plotted over time and the kinetics of viral titers and gene expression analyzed. *CCL2*, *CCL3*, *CCL4* and *CCL5* had similar expression kinetics that mirrored those of WNV titers in the tissues. Error bars represent SEM; n = 3 mice per day. * represents statistically significant changes for mRNA (magenta).

A subset of chemokine genes was then examined to assess their expression kinetics relative to viral infections in the spleen, lung, kidney and brain. Due to the fact that no infectious virus was recovered from the liver in this study, no correlations between viral infection and chemokine expression kinetics could be undertaken. Previous studies using inbred mouse strains have reproducibly shown the up-regulation of chemokines such as *Ccl2*, *Ccl3*, *Ccl4* and *Ccl5* in various tissues in response to WNV infection [37], [38], [41]–[43]. Our study also found these chemokines to be differentially expressed in various tissues. To test whether the chemokine expression kinetics correlated with viral replication kinetics, total mRNA counts of the individual genes and viral titers (PFU) over the course of infection for spleen, lung, kidney and brain were plotted. As shown in Figure 8 the expression kinetics of *Ccl2*, *Ccl3 and Ccl4* were observed to mirror the kinetics of infectious virus for the spleen (Figure 9A), lung (Figure 9B) and brain (Figure 10A) but not the kidney (Figure 10B). Expression of *Ccl5* had similar kinetics as infectious virus for spleen, lung and brain but not kidney (Figures 9 & 10).

The kinetics between peak viral infection in individual tissues and gene expression and protein expression profiles for a few select chemokines for spleen, lung, kidney and brain were investigated and similar patterns for both RNA and protein expression for all but *CCL4* in the kidney were found (data not shown).These data demonstrate the successful use of complementary high throughput gene and protein expression as reliable methods to determine specific immune responses elicited by viral infection and how these changes relate to the kinetics of viral replication.

### Specific Gene Expression Analysis of WNV infected Tissues

**Figure 11:**
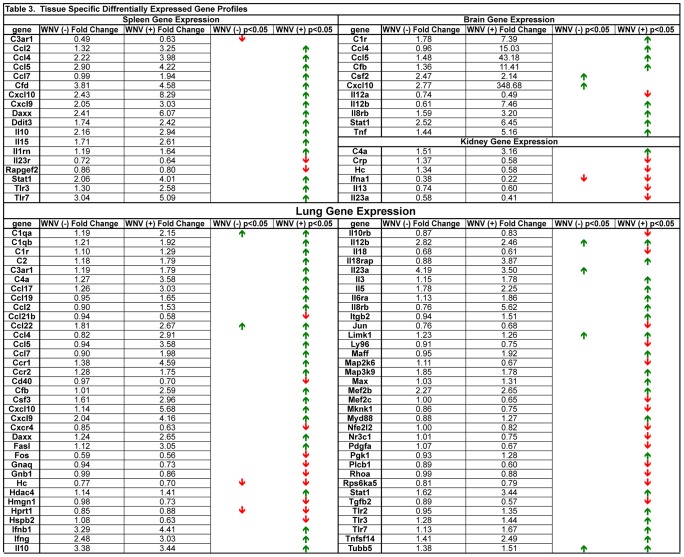
Stratification of genes that were differentially expressed in tissues determined to be infected (WNV (+)) or uninfected (WNV (−)) based on sensitive qRT-PCR detection of WNV RNA. Fold change is indicated in the columns based on mock-infected tissues. Red arrows indicate decreased gene expression and green arrows increased gene expression. Absence of arrows indicates no statistically significant change observed. Mock for all tissues n = 3; spleen WNV (−) n = 5 and WNV (+) n = 22; lung WNV (−) n = 5 and WNV (+) n = 22; liver WNV (−) n = 11 and WNV (+) n = 16; kidney WNV (+) n = 8 and WNV (+) n = 19; brain WNV (−) n = 13 and WNV (+) n = 14; p<0.05 considered significant.

The present study allowed analysis of the dynamic gene expression profiles of the inflammatory response over the course of WNV infection in outbred SW mice. Gene expression data was stratified to evaluate whether any significant changes in gene expression could be determined in tissues that were confirmed infected (WNV (+)) versus tissues that were negative for infection (WNV (−)) and PFU equivalents determined using qRT-PCR and compared both to mock-infected SW mice (Table 1). WNV (−) spleen tissue had a significantly lower *C3ar1* expression compared to WNV infected spleen (Figure 11). In WNV (−) lung, *Il23a* was significantly upregulated while *Il10rb*, an accessory protein for the *IL10* receptor, was significantly down-regulated. Genes that were significantly upregulated only in WNV (−) brain tissue were *Csf2* and *Cxcl10* (Figure 11). A large number of genes in the lung were determined to have changes in their expression in WNV (+) lung tissue vs. WNV (−) lung tissue.

**Figure 12:**
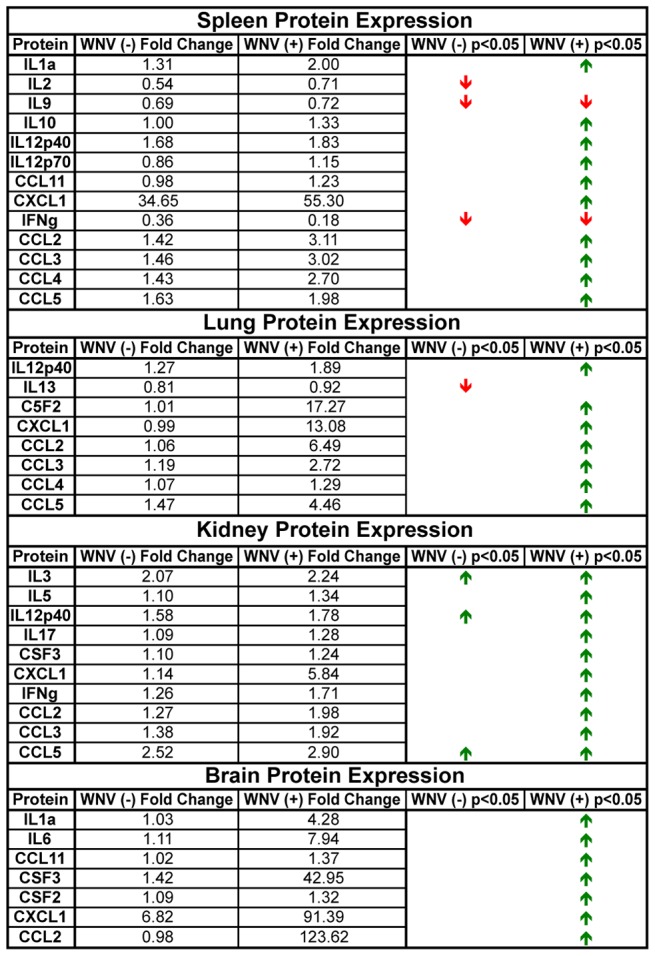
Stratification of proteins that were differentially expressed in tissues determined to be infected (WNV (+)) or uninfected (WNV (−)) based on sensitive qRT-PCR detection of WNV RNA. Fold change are indicated in the columns based on mock-infected tissues. Red arrows indicate decreased gene expression and green arrows increased gene expression. Absence of arrows indicates no statistically significant change observed. Mock for all tissues n = 3; spleen WNV (−) n = 5 and WNV (+) n = 22; lung WNV (−) n = 5 and WNV (+) n = 22; liver WNV (−) n = 11 and WNV (+) n = 16; kidney WNV (+) n = 8 and WNV (+) n = 19; brain WNV (−) n = 13 and WNV (+) n = 14; p<0.05 considered significant.

The same analysis was performed for protein expression levels using the Bio-Plex Pro data and the results are summarized in Figure 12. Overall these analyses enabled distinction between RNA and protein expression changes that may be associated with a general inflammatory response compared to genes and proteins that were specific for WNV (+) and WNV (−) tissues from WNV infected SW mice.

## Discussion

Technological advances have allowed profiling of living systems at the genome, transcriptome, and proteome levels in response to various stressors or disease states. In this study, the first in-depth characterization of WNV infection in the SW outbred weanling mouse model was performed using the nCounter platform for multiplex-gene expression analysis in a single reaction without the need of enzymatic target amplification. Unlike other platforms, (such as microarray and/or next generation sequencing) the nCounter platform enables high throughput, sensitive, quantitative, and reproducible gene expression analysis of multiple samples over the course of infection without the need for complex data analysis or confirmation of results by qRT-PCR. Thus, the nCounter enabled rapid gene expression profiling of a large number of inflammatory response genes from multiple tissues over the course of WNV infection in an outbred mouse model and to rapidly differentiate between general versus specific gene expression changes in WNV (+) vs. WNV (−) tissues.

The temporal and dynamic analyses of viral burdens, kinetics and tissue tropisms generated results similar to those described for WNV infection using inbred mouse models, such as the C3H/HeN, C57BL/6 and BABL/C (for extensive reviews see [12], [13]). Therefore, despite the high susceptibility of SW mice to neuroinvasive WNV disease, their responses to infection are generally comparable to those of more resistant inbred strains such as C57BL/6. Following peak viremia, WNV dissemination to secondary tissues was observed in the spleen, lung, kidney, and brain of infected SW outbred mice. Although the animals used in these studies were not perfused prior to collection of tissues, we assumed some of the infectious virus detectable at days 1 to 3 could be present from blood. However, in all cases but the liver the tissue titers were high enough (equal to or greater than the concurrent PFU/mL virus titer in serum) to suggest local replication was occurring. Overall, these studies demonstrated similar viral kinetics and tissue tropisms in SW outbred mice and inbred mice used to study WNV infection. Brown *et al* [9] described differences in survival rates of 6-week-old C3H and C57BL/6 mice despite comparable levels of neuroinvasion and similar virus loads in the periphery. Use of the nCounter system, coupled with other genomic and proteomic tools, in focused comparative studies between mouse strains and assessing other host variables that appear to influence susceptibility, such as age, should provide novel insights into specific responses that correlate with protection against neuroinvasion and/or lethal WNV disease following peripheral infection.

Infection of the lung had only been previously described in wild-type (WT) C3H/HeN and C57BL/6 [9] and in *IFN-α/βR^−/−^* mice [17]. Relevant to this observation, recent studies found a strong correlation between respiratory insufficiency and mortality in WNV infected hamsters where infection of the ventrolateral medulla, responsible for respiratory control, was reported [44]. In this study, DEG profiles of the lung revealed an increase in expression for *TLR2*, *TLR3*, *TLR6* and *TLR7* genes. Previous studies in *TLR3^−/−^* mice have shown increased susceptibility and resistance to WNV infection [45], [46]. *TLR7* and the adaptor protein MYD88 have been shown to be required against lethal WNV infection [40], [47]. In other systems *TLR2* signaling has been shown to be activated by the hemagglutinin protein of measles virus [48], Core/NS3 protein of HCV [49] and in mice infected with HCMV, LCMV [50], [51] and HSV-1 [52]. Interestingly a recent study on influenza demonstrated TLR2/6 signaling to induce a protective role in mice [53]. Whether this increased expression of TLR2 and TLR6 mediates protective immunity against WNV infection in the lung remains to be further studied.

In the context of WNV infection, the liver is generally considered to be non-or poorly permissive to WNV infection [9]. Although the studies presented here demonstrated detection of genomic WNV RNA using qRT-PCR despite lack of recovery of infectious virus from this tissue at any timepoint, it is unclear whether this represents a low level of viral replication in this organ. Similar to this observation, in another recent study, the livers of WT C57BL/6 infected subcutaneously with a Texas 2002 WNV strain (lineage I) were shown to be infected using sensitive qRT-PCR but not plaque assay [31]. The data presented here also indicate that expression of complement factors during WNV infection in SW mice is tissue specific, and suggest complement expression in the liver to be the result of a systemic acute phase response to WNV infection and not necessarily the result of infection of the liver.

In the kidney, chemoattractant genes *CXCL2*, *CXCL3* and *CCL22* were down-regulated. Previous studies have shown that polymorphonuclear leukocytes have increased *CXCL1* and *CXCL2* expression when infected with WNV [54]. Interestingly, this study also found increased expression of *CXCL1*, *CCL2*, *CCL3* and *CCL5* proteins in WNV-infected kidney tissue. Expression of locally secreted chemokines has been shown to aid in the recruitment of leukocytes in the initiation and amplification phase of renal inflammation. However, these can also cause renal damage through the release of pro-inflammation and pro-fibrotic factors [55]. Thus the rapid down modulation of these genes can serve to limit acute inflammation and prevent renal damage. Whether the decreased expressions in these studies are associated with this balance response remains to be determined.

Consistent with earlier studies in inbred mouse strains, DEG that were identified in the spleen, lung, kidney, liver and brain were primarily associated with chemokine, cytokine, complement, cytoskeletal and signal transduction, pathogen-associate molecular patterns and transcriptional regulator functions [9], [26], [30], [31]. KEGG pathway enrichment analysis of DEG using DAVID also helped identify chemokine/cytokine interactions [34], [56], Toll-like receptor signaling [57]–[60], NOD-like receptor signaling [13], [57], [61] and complement and coagulation pathways [13], [18] which are known to control WNV infection in inbred mouse models. Data stratification of infected vs. non-infected tissues from WNV infected SW mouse allowed differentiation of gene expression responses that we considered to be a general inflammatory response to WNV infection versus tissue specific responses due to infection with WNV. Complementary high throughput protein analysis enabled gene and protein expression kinetics that serve to further validate the gene expression analysis alone.

The nCounter system and complementary methods employed here provide a powerful platform for detailed comparative analysis of the kinetics and magnitude of host responses to WNV infection. Gene expression profiling using the nCounter system provided a rapid and sensitive platform to follow dynamic gene expression changes through the course of WNV infection. Future applications for this technology include comparable studies using variably attenuated WNV strains in the SW mouse model, or virulent WNV strains such as NY99 in more resistant mouse models, to comprehensively define specific innate and adaptive immune responses associated with protection and immunity for the purpose of designing and testing of antiviral therapies and vaccines against WNV infection and neuroinvasive disease. Finally, similar studies using other flaviviruses in similar or different animal models should enhance understanding of host responses common to infection by these viruses.

## Supporting Information

Figure S1 Volcano plot of changes in Gene Expression in WNV infected SW outbred Mice. Gene expression profiles for spleen, lung, liver, kidney and brain were analyzed over the course on infection. Changes in gene expression profiles for each tissue was plotted on the Y-axis based on statistical significance in reverse order and fold change (log2) was plotted on the X-axis. Data points represent n = 3 mice per day, p<0.05 considered statistically significant.
(TIFF)

Figure S2 Protein-protein interaction networks identified for all differentially expressed genes in (A) spleen, (B) kidney, (C) lung, (D) liver, and (E) brain using the search tool for the retrieval of interacting genes/proteins (STRING). Confidence view of protein-protein interaction network from DEGs from the liver of WNV infected SW mice. Network clustering was performed with Kmeans = 5. The thickness of the blue line connecting genes and nodes indicates the confidence score of association identified.
(SVG)

Figure S3 Confidence view of cytokine-cytokine receptor interactions in the lung protein-protein interaction network of WNV-infected SW mice. Network clustering was performed with Kmeans = 2. The thickness of the blue line connecting genes and nodes indicates the confidence score of association identified.
(TIF)

Figure S4 Confidence view of cytokine-cytokine receptor interactions in the liver protein-protein interaction network of WNV-infected SW mice. Network clustering was performed with Kmeans = 2. The thickness of the blue line connecting genes and nodes indicates the confidence score of association identified.
(TIF)

Figure S5 Confidence view of cytokine-cytokine receptor interactions in the spleen protein-protein interaction network of WNV-infected SW mice. Network clustering was performed with Kmeans = 2. The thickness of the blue line connecting genes and nodes indicates the confidence score of association identified.
(TIF)

Figure S6 Confidence view of cytokine-cytokine receptor interactions in the kidney protein-protein interaction network of WNV-infected SW mice. Network clustering was performed with Kmeans = 2. The thickness of the blue line connecting genes and nodes indicates the confidence score of association identified.
(TIF)

Figure S7 Confidence view of Toll-like receptor signaling pathways interactions in the lung protein-protein interaction network of WNV-infected SW mice. Network clustering was performed with Kmeans = 2. The thickness of the blue line connecting genes and nodes indicates the confidence score of association identified.
(TIF)

Figure S8 Confidence view of Toll-like receptor signaling pathways interactions in the spleen protein-protein interaction network of WNV-infected SW mice. Network clustering was performed with Kmeans = 2. The thickness of the blue line connecting genes and nodes indicates the confidence score of association identified.
(TIF)

Figure S9 Confidence view of complement and coagulation cascade interactions in the liver protein-protein interaction network of WNV-infected SW mice. Network clustering was performed with Kmeans = 2. The thickness of the blue line connecting genes and nodes indicates the confidence score of association identified.
(TIF)

Figure S10 Confidence view of JAK-STAT signaling pathway interactions in the kidney protein-protein interaction network of WNV-infected SW mice. Network clustering was performed with Kmeans = 2. The thickness of the blue line connecting genes and nodes indicates the confidence score of association identified.
(TIF)

Table S1 List of genes for which mRNA was quantitated using the Nanostring nCounter system in this study with corresponding accession numbers.
(PDF)
