# Supplementary figures and images for: Multiplexed Digital mRNA Profiling of the Inflammatory Response in the West Nile Swiss Webster Mouse Model
Source: PLoS Negl Trop Dis. 2014 Oct 23;8(10):e3216. doi: 10.1371/journal.pntd.0003216 (PMC4207670; doi:10.1371/journal.pntd.0003216)

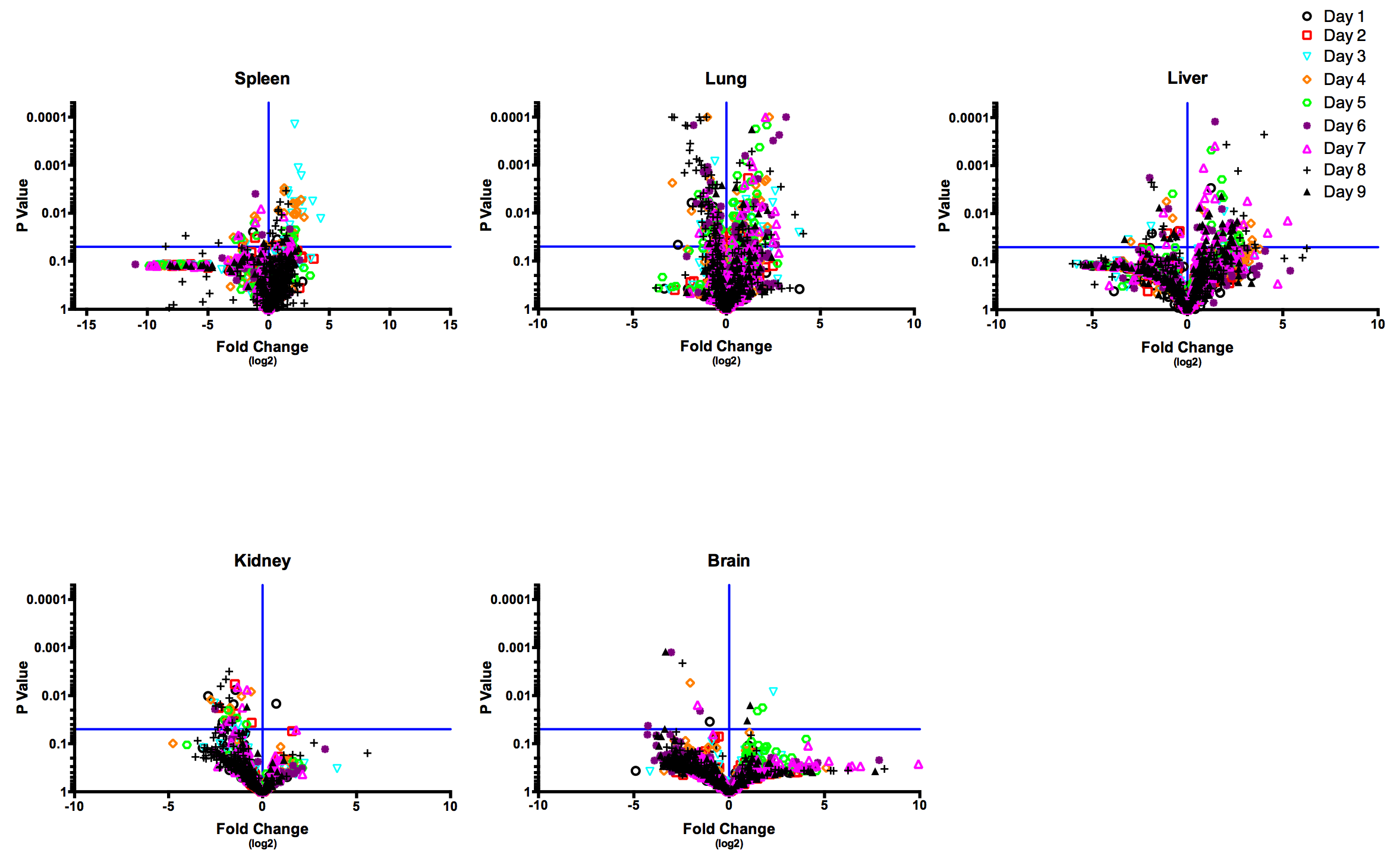

Supplement: Figure S1 — Volcano plot of changes in Gene Expression in WNV infected SW outbred Mice. Gene expression profiles for spleen, lung, liver, kidney and brain were analyzed over the course on infection. Changes in gene expression profiles for each tissue was plotted on the Y-axis based on statistical significance in reverse order and fold change (log2) was plotted on the X-axis. Data points represent n = 3 mice per day, p<0.05 considered statistically significant. (TIFF) [file pntd.0003216.s001.tiff]

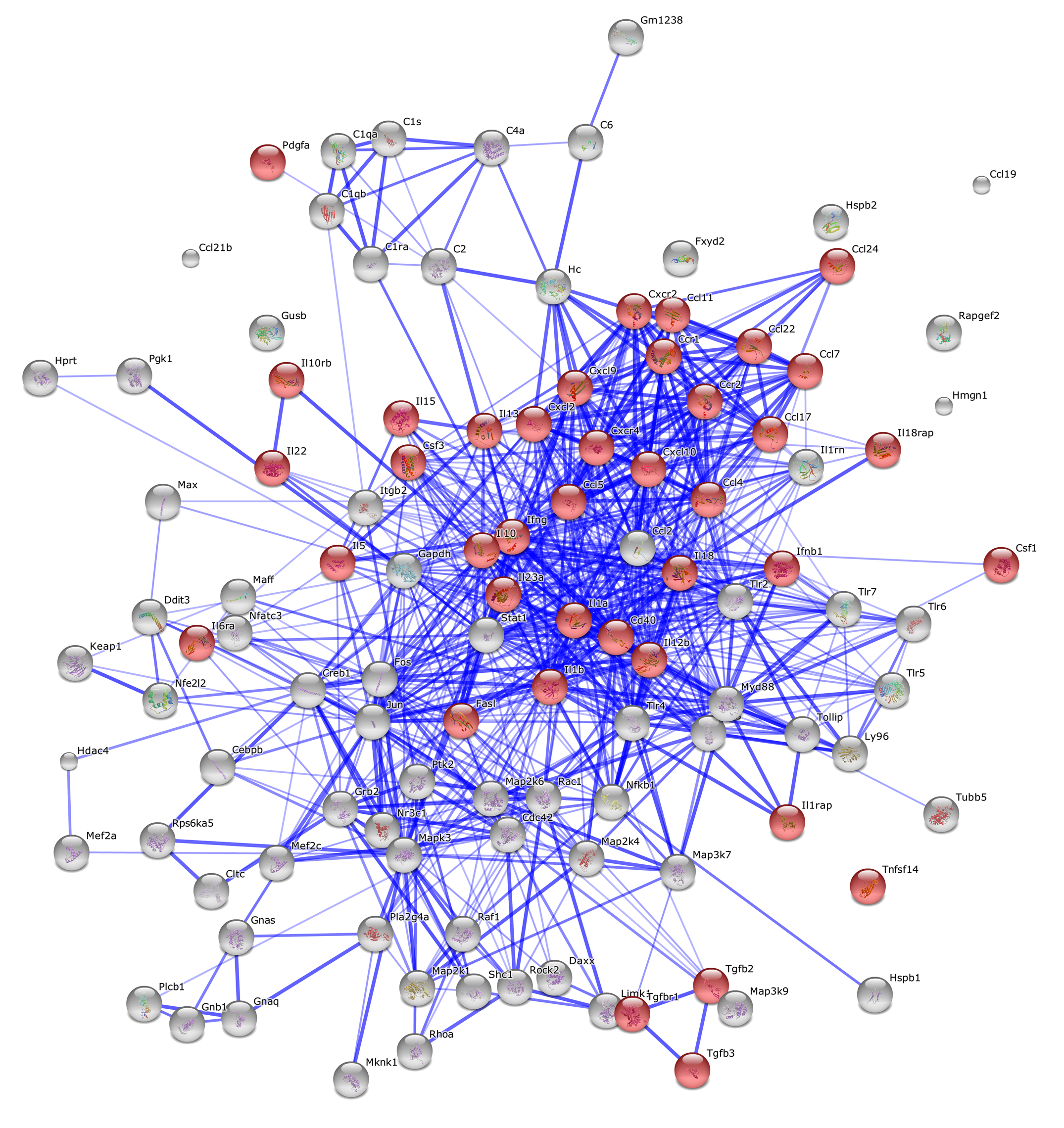

Supplement: Figure S3 — Confidence view of cytokine-cytokine receptor interactions in the lung protein-protein interaction network of WNV-infected SW mice. Network clustering was performed with Kmeans = 2. The thickness of the blue line connecting genes and nodes indicates the confidence score of association identified. (TIF) [file pntd.0003216.s003.tif]

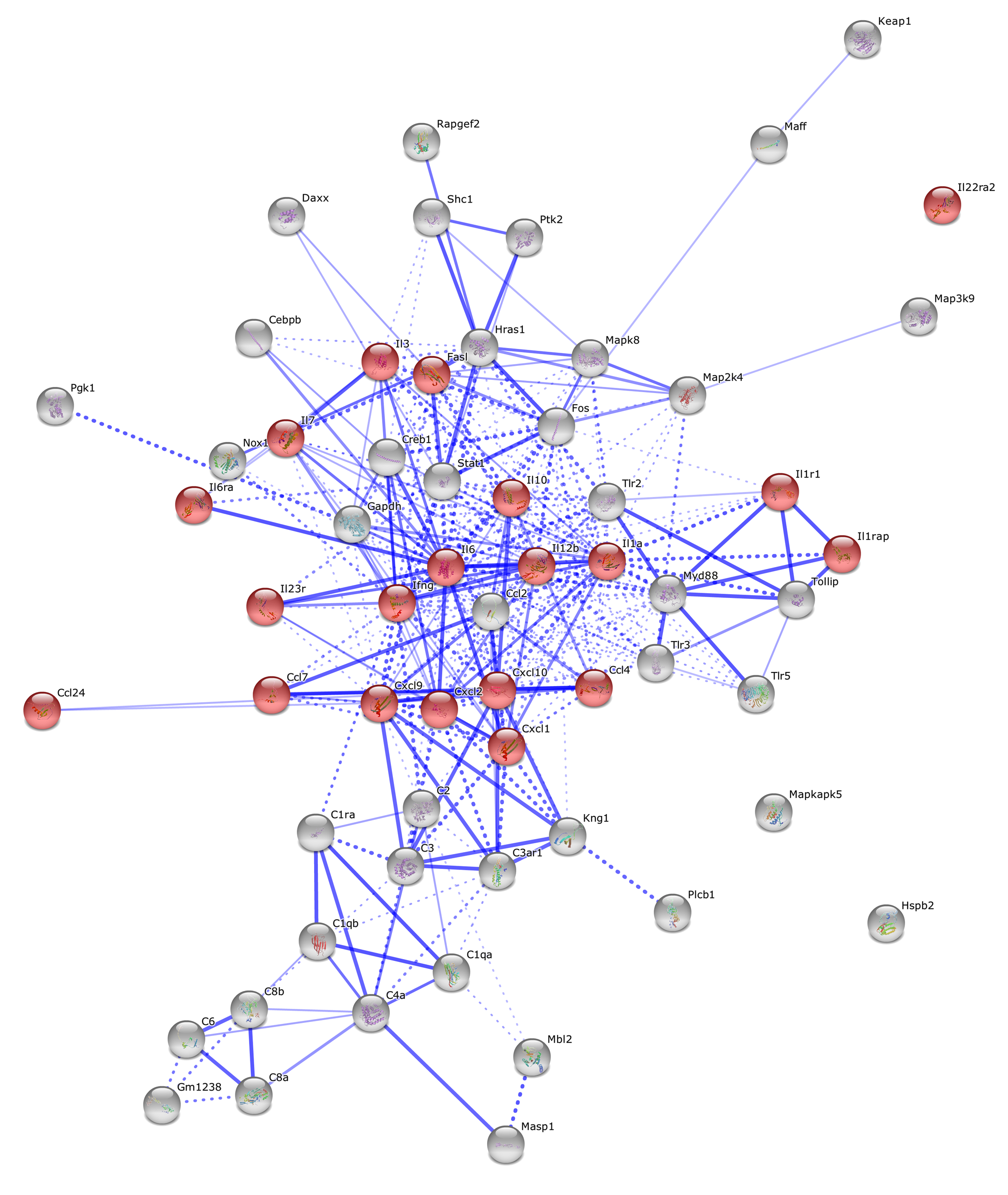

Supplement: Figure S4 — Confidence view of cytokine-cytokine receptor interactions in the liver protein-protein interaction network of WNV-infected SW mice. Network clustering was performed with Kmeans = 2. The thickness of the blue line connecting genes and nodes indicates the confidence score of association identified. (TIF) [file pntd.0003216.s004.tif]

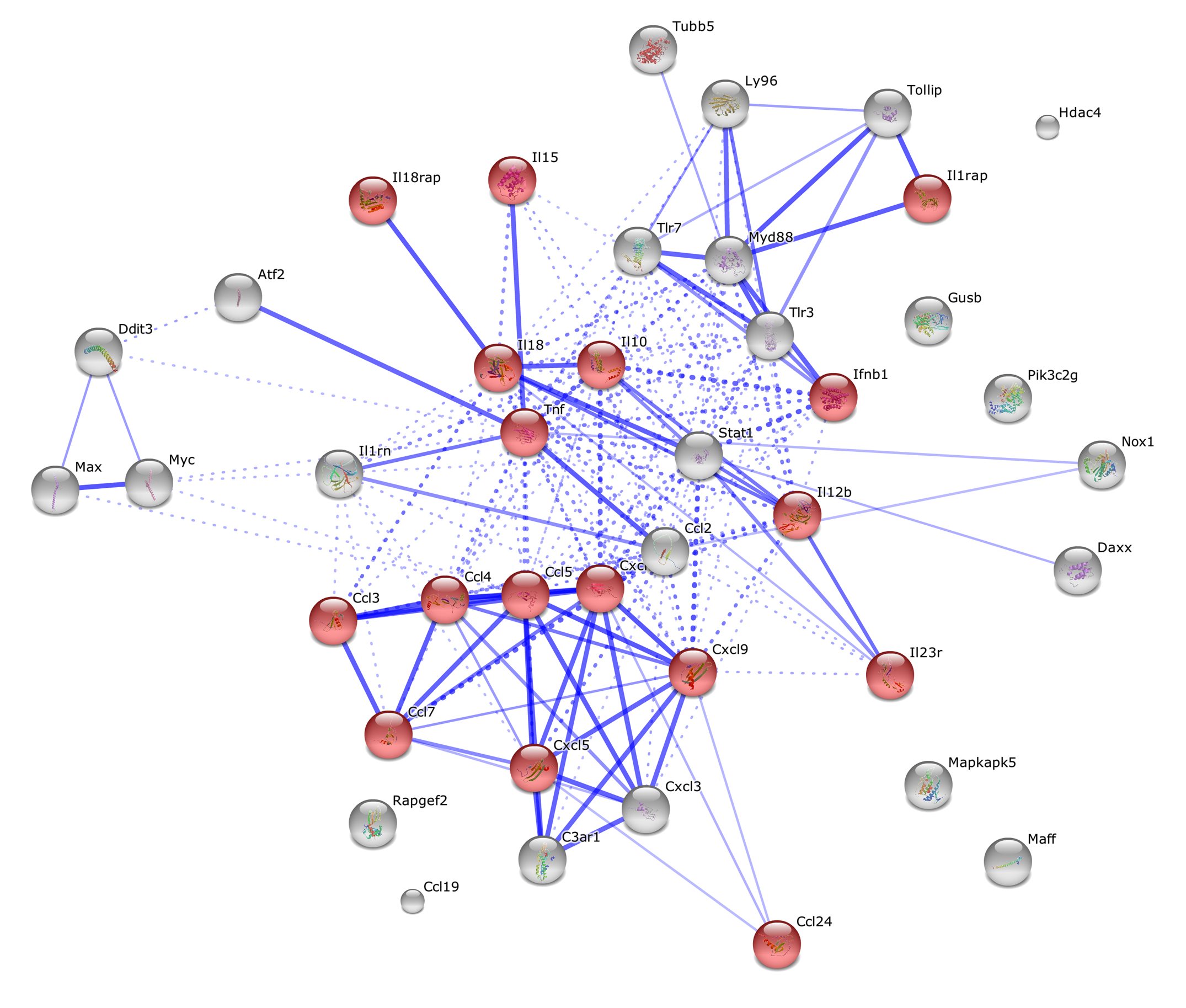

Supplement: Figure S5 — Confidence view of cytokine-cytokine receptor interactions in the spleen protein-protein interaction network of WNV-infected SW mice. Network clustering was performed with Kmeans = 2. The thickness of the blue line connecting genes and nodes indicates the confidence score of association identified. (TIF) [file pntd.0003216.s005.tif]

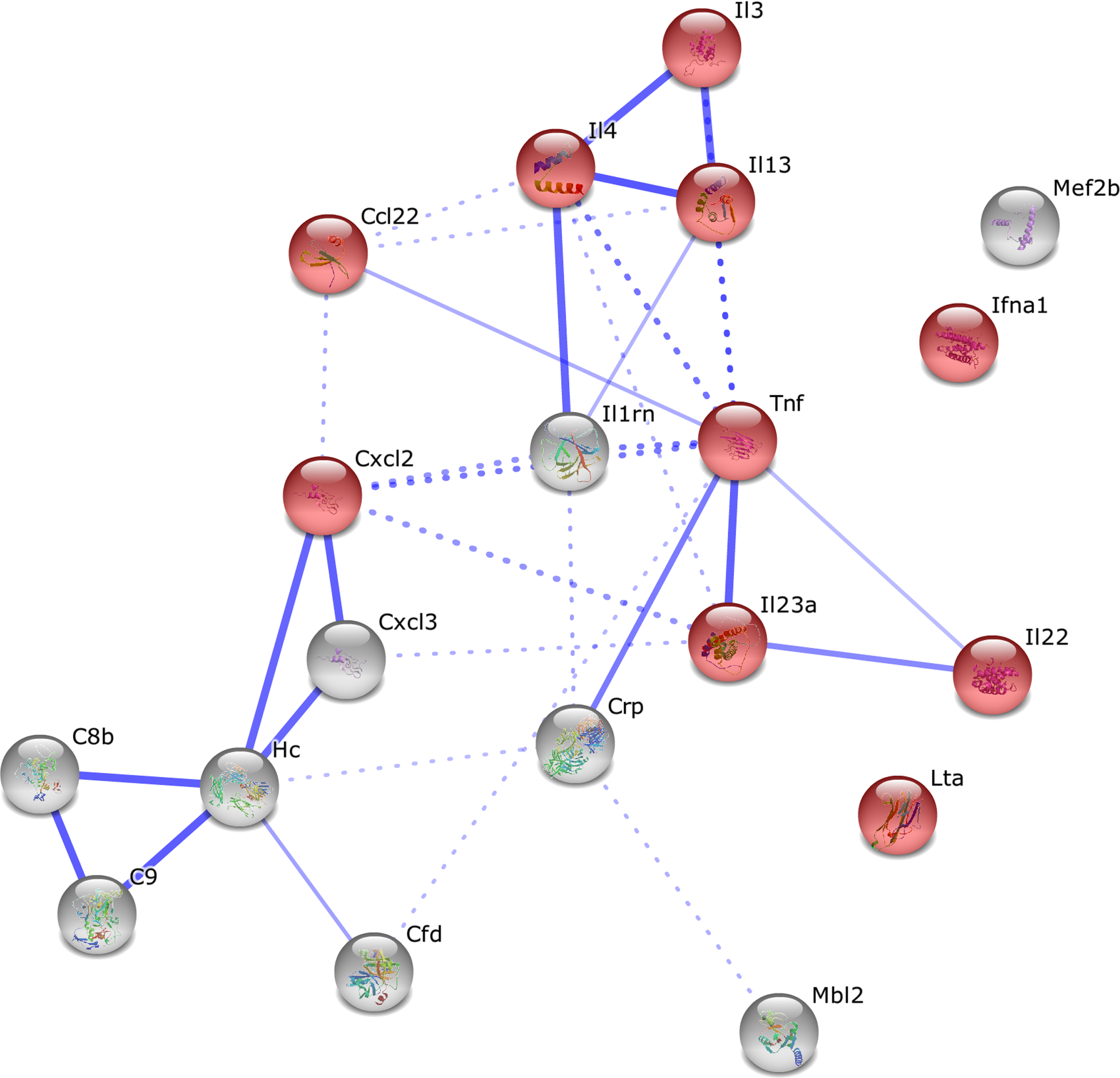

Supplement: Figure S6 — Confidence view of cytokine-cytokine receptor interactions in the kidney protein-protein interaction network of WNV-infected SW mice. Network clustering was performed with Kmeans = 2. The thickness of the blue line connecting genes and nodes indicates the confidence score of association identified. (TIF) [file pntd.0003216.s006.tif]

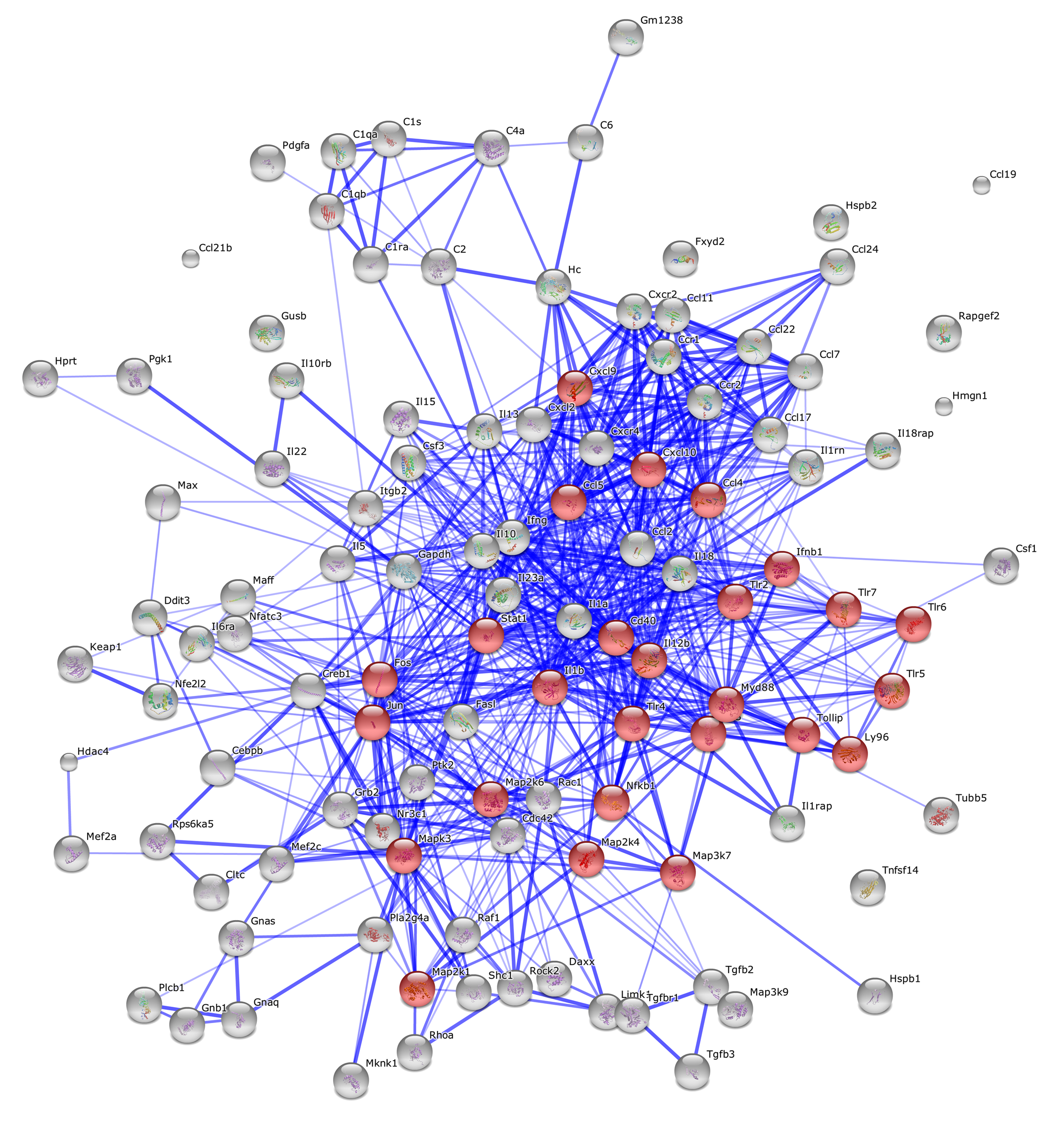

Supplement: Figure S7 — Confidence view of Toll-like receptor signaling pathways interactions in the lung protein-protein interaction network of WNV-infected SW mice. Network clustering was performed with Kmeans = 2. The thickness of the blue line connecting genes and nodes indicates the confidence score of association identified. (TIF) [file pntd.0003216.s007.tif]

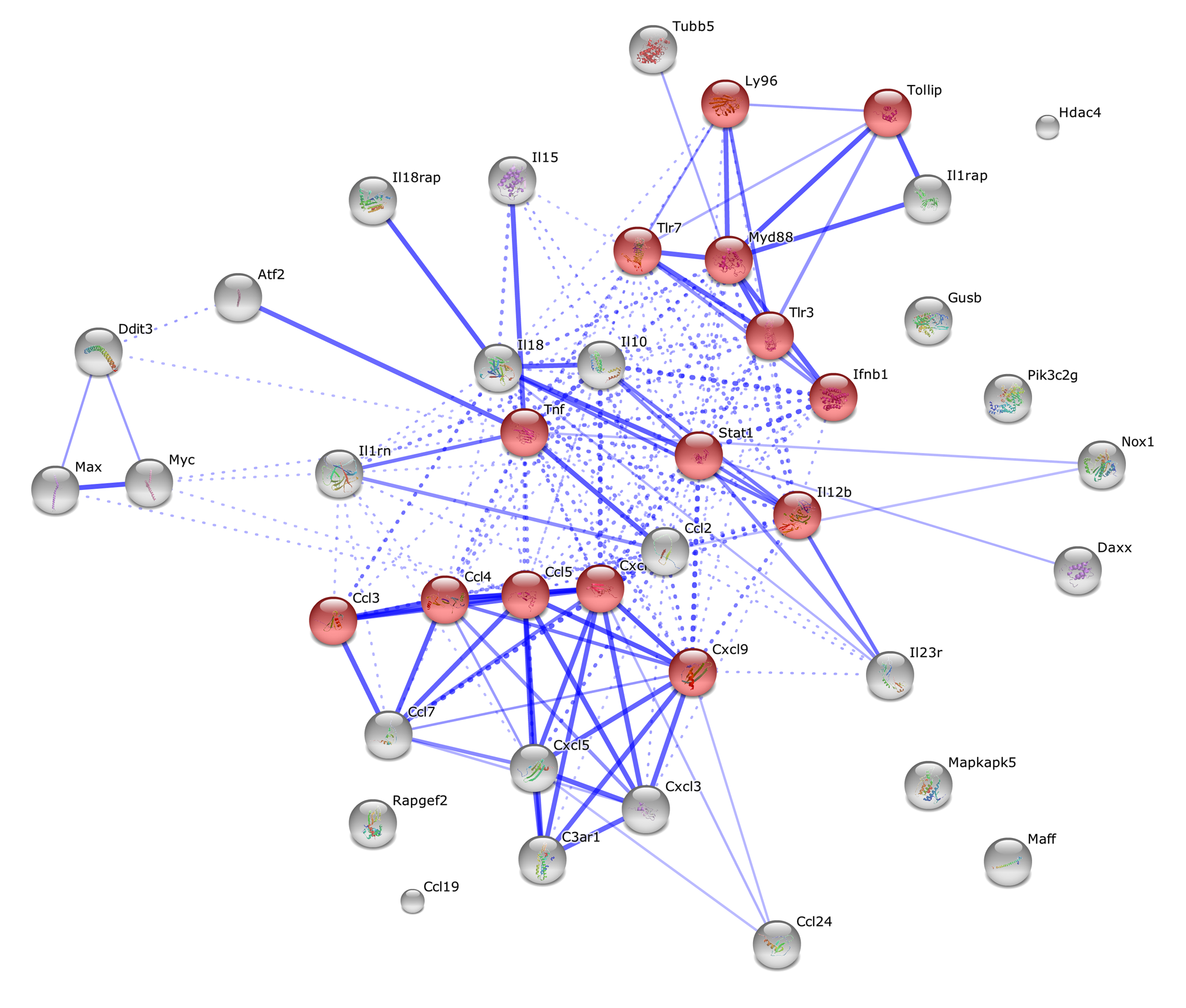

Supplement: Figure S8 — Confidence view of Toll-like receptor signaling pathways interactions in the spleen protein-protein interaction network of WNV-infected SW mice. Network clustering was performed with Kmeans = 2. The thickness of the blue line connecting genes and nodes indicates the confidence score of association identified. (TIF) [file pntd.0003216.s008.tif]

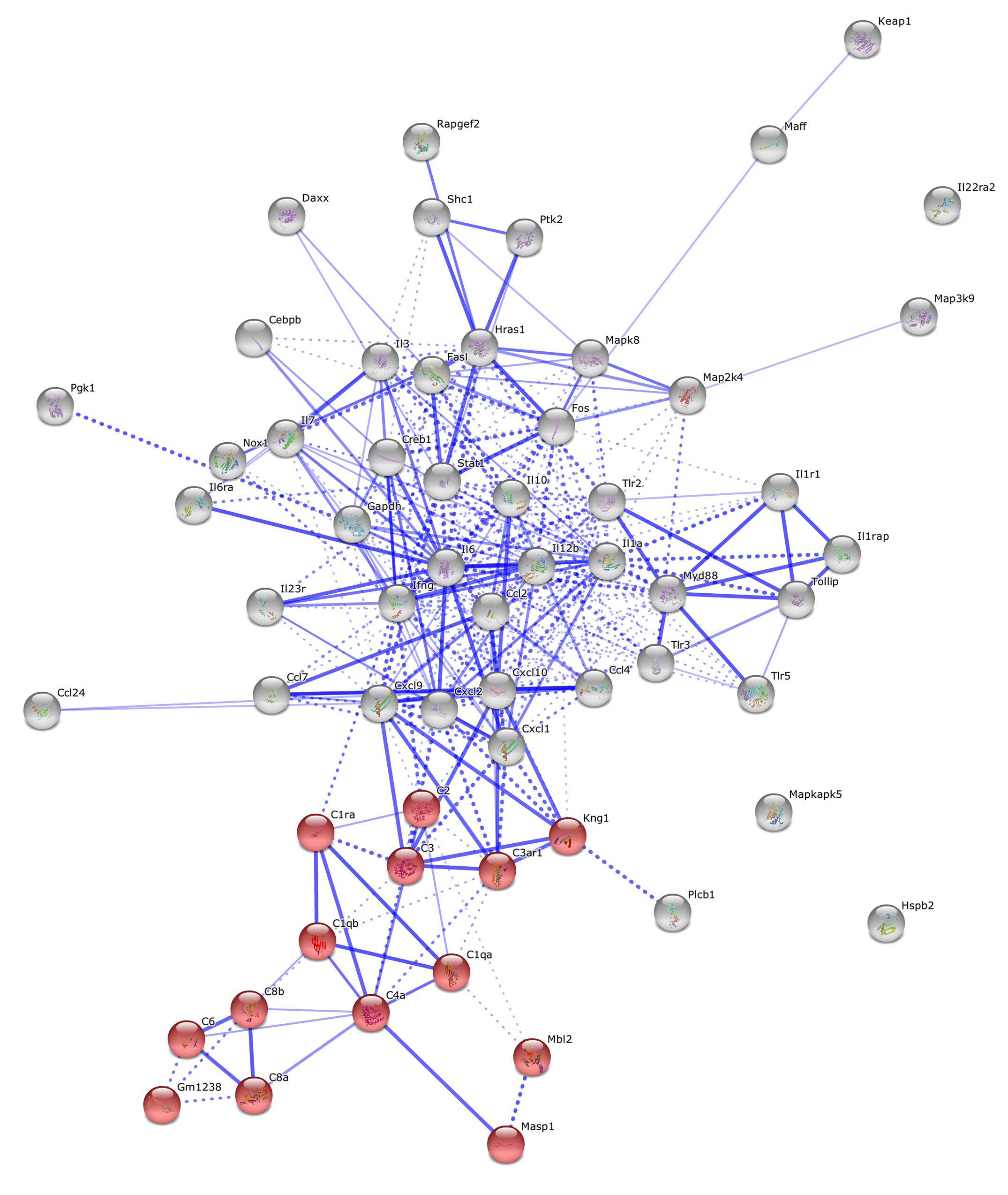

Supplement: Figure S9 — Confidence view of complement and coagulation cascade interactions in the liver protein-protein interaction network of WNV-infected SW mice. Network clustering was performed with Kmeans = 2. The thickness of the blue line connecting genes and nodes indicates the confidence score of association identified. (TIF) [file pntd.0003216.s009.tif]

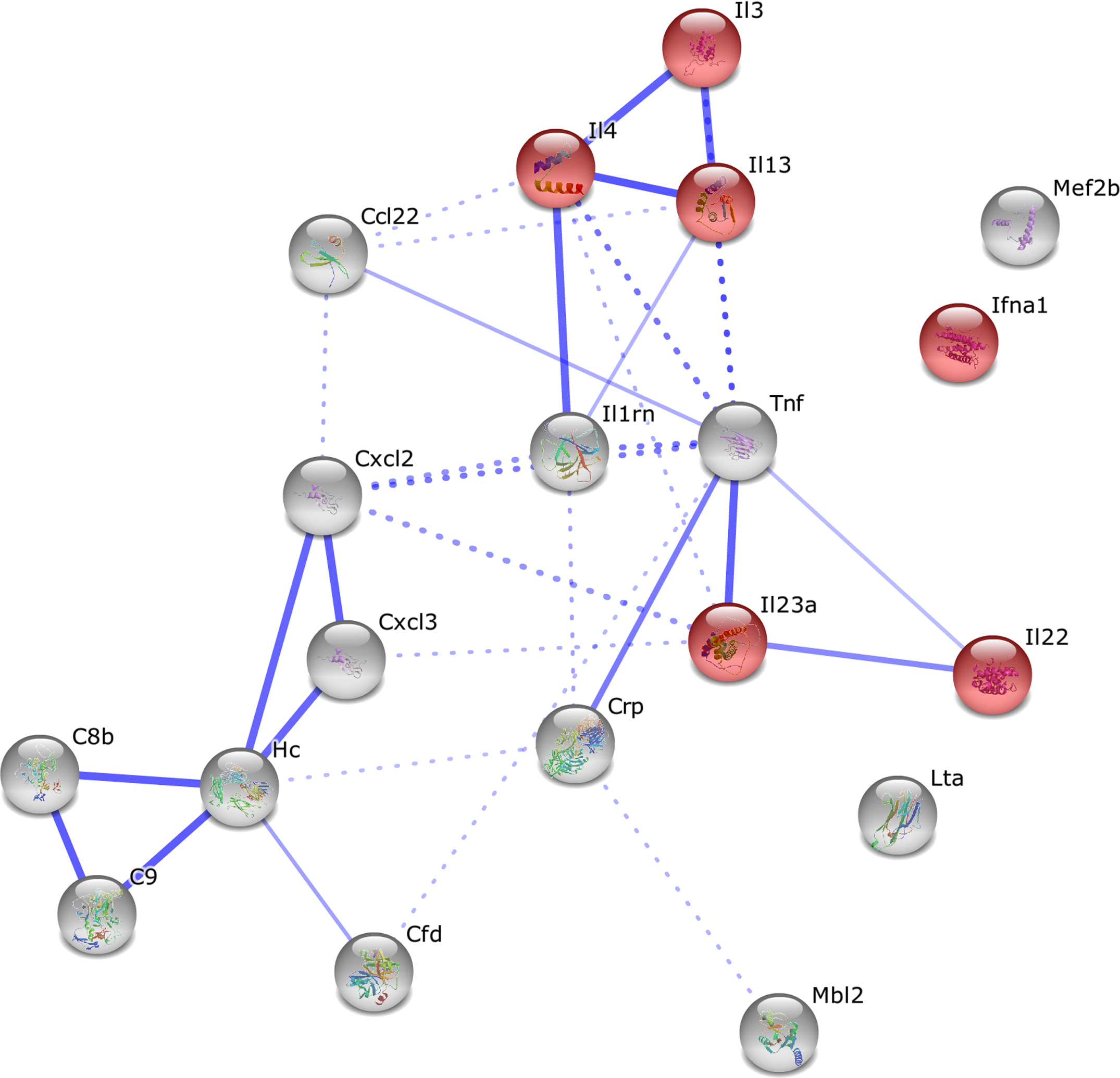

Supplement: Figure S10 — Confidence view of JAK-STAT signaling pathway interactions in the kidney protein-protein interaction network of WNV-infected SW mice. Network clustering was performed with Kmeans = 2. The thickness of the blue line connecting genes and nodes indicates the confidence score of association identified. (TIF) [file pntd.0003216.s010.tif]
