# Supplementary material for: Multiplexed Digital mRNA Profiling of the Inflammatory Response in the West Nile Swiss Webster Mouse Model
Source: PLoS Negl Trop Dis. 2014 Oct 23;8(10):e3216. doi: 10.1371/journal.pntd.0003216 (PMC4207670; doi:10.1371/journal.pntd.0003216)
Supplement: Table S1 — List of genes for which mRNA was quantitated using the Nanostring nCounter system in this study with corresponding accession numbers. (PDF) [file pntd.0003216.s011.pdf]

Table S1: List of genes analyzed in this study with accession numbers

Gene	Accession #	Gene	Accession #	Gene	Accession #
Atf2	NM_001025093.1	Ccr2	NM_009915.2	Fasl	NM_010177.3
Bcl6	NM_009744.3	Ccr3	NM_009914.4	Fos	NM_010234.2
C1qa	NM_007572.2	Ccr4	NM_009916.2	Fxyd2	NM_052823.2
C1qb	NM_009777.2	Ccr7	NM_007719.2	Gnaq	NM_008139.5
C1r	NM_023143.3	Cd4	NM_013488.2	Gnas	NM_010309.3
C1s	NM_144938.2	Cd40	NM_011611.2	Gnb1	NM_008142.3
C2	NM_013484.2	Cd40lg	NM_011616.2	Gngt1	NM_010314.2
C3	NM_009778.2	Cd55	NM_010016.2	Grb2	NM_008163.3
C3ar1	NM_009779.2	Cdc42	NM_009861.1	H2-Ea	NM_010381.2
C4a	NM_011413.2	Cebpb	NM_009883.3	H2-Eb1	NM_010382.2
C6	NM_016704.2	Cfb	NM_008198.2	Hc	NM_010406.1
C7	XM_356827.6	Cfd	NM_013459.1	Hdac4	NM_207225.1
C8a	NM_146148.1	Cfl1	NM_007687.2	Hmgn1	NM_008251.3
C8b	NM_133882.2	Creb1	NM_133828.2	Hras1	NM_008284.2
C9	NM_013485.1	Crp	NM_007768.4	Hspb1	NM_013560.2
Ccl11	NM_011330.3	Csf1	NM_001113530.1	Hspb2	NM_024441.2
Ccl17	NM_011332.2	Csf2	NM_009969.4	Ifna1	NM_010502.2
Ccl19	NM_011888.2	Csf3	NM_009971.1	Ifnb1	NM_010510.1
Ccl2	NM_011333.3	Cxcl1	NM_008176.1	Ifng	NM_008337.1
Ccl21b	NM_011124.4	Cxcl10	NM_021274.1	Il10	NM_010548.1
Ccl22	NM_009137.2	Cxcl2	NM_009140.2	Il10rb	NM_008349.5
Ccl24	NM_019577.4	Cxcl3	NM_203320.2	Il11	NM_008350.2
Ccl3	NM_011337.1	Cxcl5	NM_009141.2	Il12a	NM_008351.1
Ccl4	NM_013652.1	Cxcl9	NM_008599.2	Il12b	NM_008352.1
Ccl5	NM_013653.1	Cxcr4	NM_009911.3	Il13	NM_008355.2
Ccl7	NM_013654.2	Daxx	NM_007829.3	Il15	NM_008357.1
Ccl8	NM_021443.2	Ddit3	NM_007837.3	Il18	NM_008360.1
Ccr1	NM_009912.4	Elk1	NM_007922.4	Il18rap	NM_010553.2

Il1a	NM_010554.4	Map2k1	NM_008927.3	Nr3c1	NM_008173.3
Il1b	NM_008361.3	Map2k4	NM_009157.4	Pdgfa	NM_008808.3
Il1r1	NM_001123382.1	Map2k6	NM_011943.2	Pik3c2g	NM_011084.2
Il1rap	NM_008364.2	Map3k1	NM_011945.2	Pla2g4a	NM_008869.2
Il1rn	NM_031167.4	Map3k5	NM_008580.4	Plcb1	NM_019677.1
Il2	NM_008366.2	Map3k7	NM_172688.2	Ppp1r12b	NM_001081307.1
Il22	NM_016971.1	Map3k9	NM_177395.4	Prkca	NM_011101.3
Il22ra2	NM_178258.5	Mapk1	NM_001038663.1	Prkcb1	NM_008855.2
Il23a	NM_031252.1	Mapk14	NM_011951.2	Ptk2	NM_007982.2
Il23r	NM_144548.1	Mapk3	NM_011952.2	Rac1	NM_009007.2
Il3	NM_010556.4	Mapk8	NM_016700.3	Raf1	NM_029780.3
Il4	NM_021283.1	Mapkapk2	NM_008551.1	Rapgef2	NM_001099624.2
Il5	NM_010558.1	Mapkapk5	XM_990515.1	Rhoa	NM_016802.4
Il6	NM_031168.1	Masp1	NM_008555.2	Ripk1	NM_009068.3
Il6ra	NM_010559.2	Masp2	NM_010767.3	Ripk2	NM_138952.3
Il7	NM_008371.2	Max	NM_008558.1	Rock2	NM_009072.2
Il8ra	NM_178241.4	Mbl2	NM_010776.1	Rps6ka5	NM_153587.2
Il8rb	NM_009909.3	Mef2a	XM_976032.1	Shc1	NM_011368.4
Il9	NM_008373.1	Mef2b	NM_001045484.1	Stat1	NM_009283.3
Itgb2	NM_008404.4	Mef2c	NM_025282.2	Tgfb1	NM_011577.1
Jun	NM_010591.2	Mef2d	NM_133665.3	Tgfb2	NM_009367.1
Keap1	NM_016679.4	Mknk1	NM_021461.4	Tgfb3	NM_009368.2
Kng1	NM_023125.3	Myc	NM_010849.4	Tgfbr1	NM_009370.2
Limk1	NM_010717.2	Myd88	NM_010851.2	Tlr1	NM_030682.1
Lta	NM_010735.1	Myl2	NM_010861.3	Tlr2	NM_011905.2
Ltb	NM_008518.2	Nfatc3	NM_010901.2	Tlr3	NM_126166.2
Ly96	NM_016923.1	Nfe2l2	NM_010902.3	Tlr4	NM_021297.2
Maff	NM_010755.3	Nfkb1	NM_008689.2	Tlr5	NM_016928.2
Mafg	XM_001002362.1	Nos2	NM_010927.3	Tlr6	NM_011604.3
Mafk	NM_010757.2	Nox1	NM_172203.1	Tlr7	NM_133211.3

Tnf	NM_013693.1				
Tnfsf14	NM_019418.2				
Tollip	NM_023764.3				
Tradd	NM_001033161.2				
Traf2	NM_009422.2				
